# Supplementary figures and images for: A novel virtual screening procedure identifies Pralatrexate as inhibitor of SARS-CoV-2 RdRp and it reduces viral replication in vitro
Source: PLoS Comput Biol. 2020 Dec 31;16(12):e1008489. doi: 10.1371/journal.pcbi.1008489 (PMC7774833; doi:10.1371/journal.pcbi.1008489)

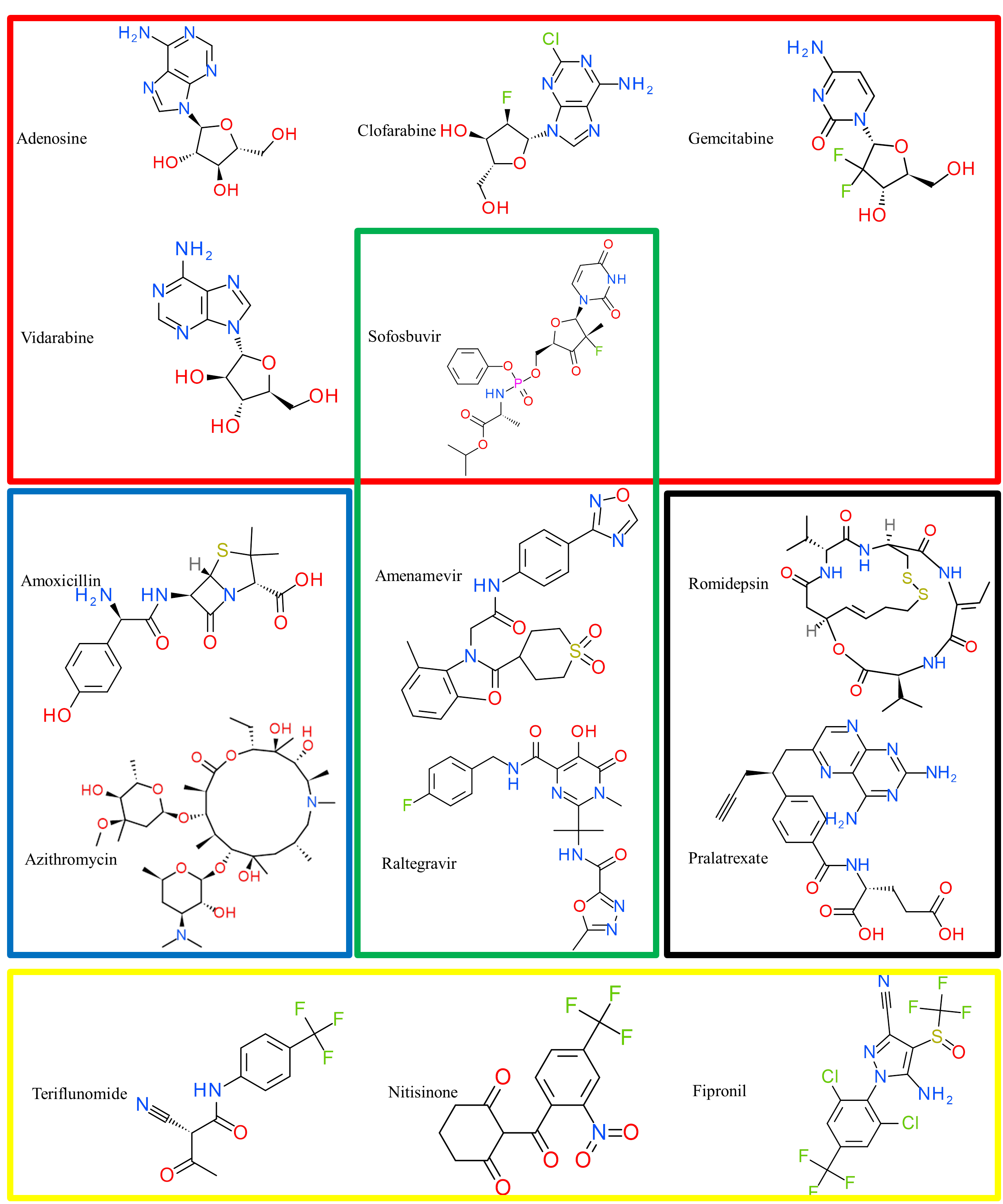

Supplement: S1 Fig — The 5 compounds in the red box belong to nucleoside analogue; the 2 compounds in the blue box are antibiotic drugs; the 3 compounds in the green box are known antivirus drugs; the 2 compounds within the black box are known anticancer drugs; the 3 compounds in the yellow box are other types. (TIF) [file pcbi.1008489.s001.tif]

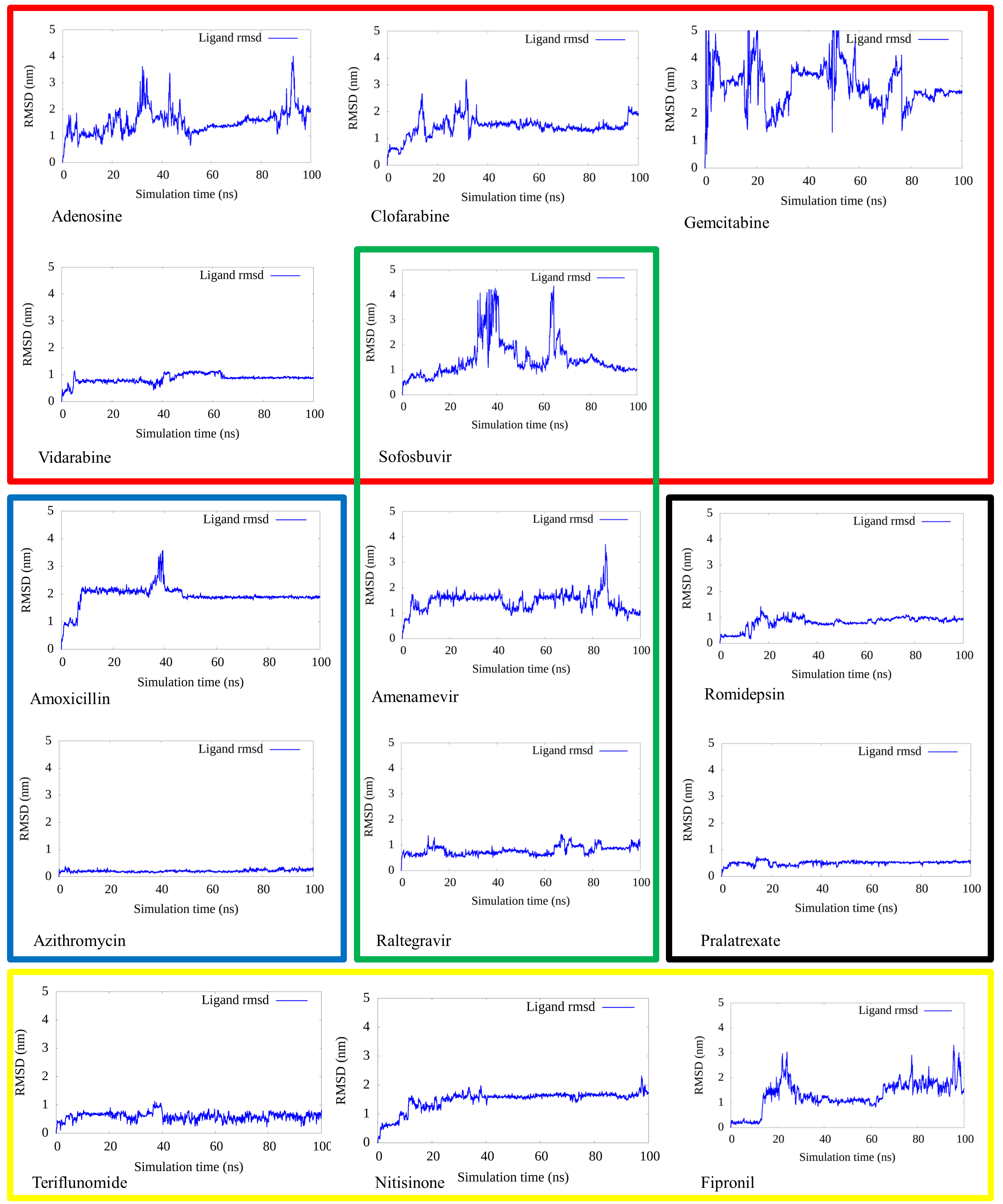

Supplement: S2 Fig — The meaning of color boxes is the same as S1 Fig. (TIF) [file pcbi.1008489.s002.tif]

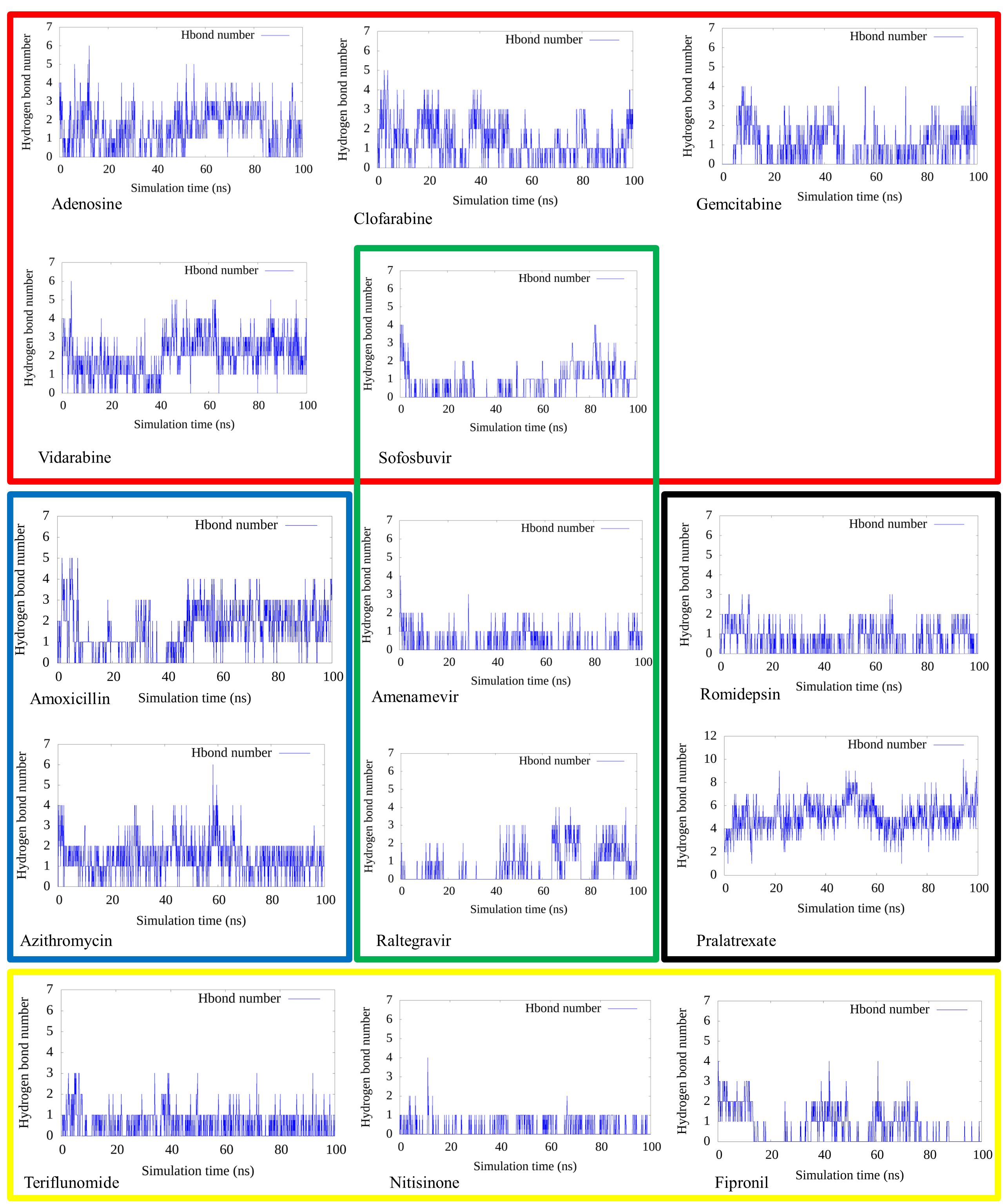

Supplement: S3 Fig — The meaning of color boxes is the same as S1 Fig. (TIF) [file pcbi.1008489.s003.tif]

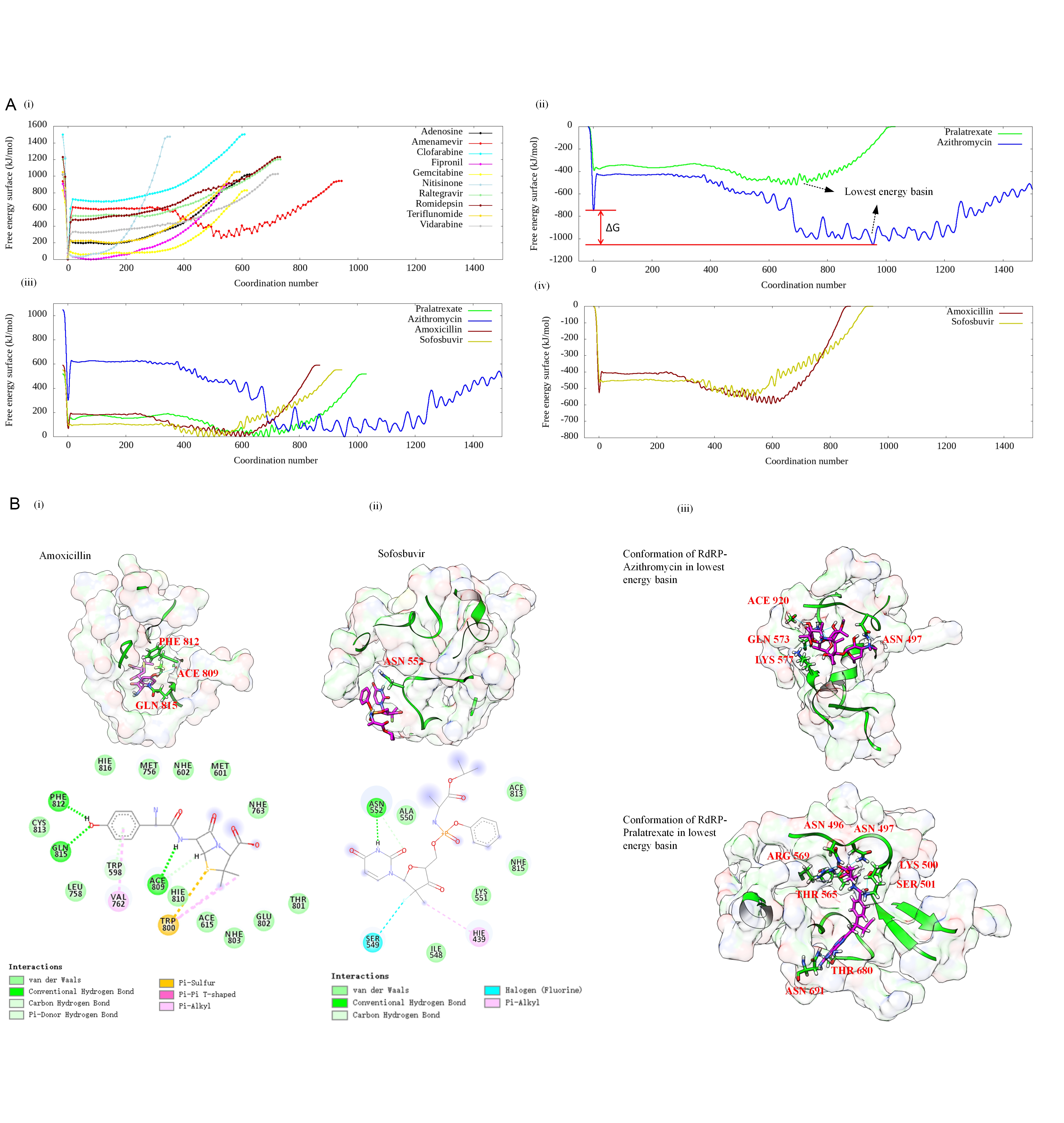

Supplement: S4 Fig — A, 1D free energy vs coordination number as CV (collective variable) from Metadynamics simulations; (i) and (iii), lowest free energy are shifted to 0 for comparison. (i) 10 drugs have much less interactions with RdRp because their lowest energy basins are near coordination number 0. (iii) The RdRp-drug complex structures in lowest energy basins of 4 drug candidates (Amoxicillin, Azithromycin, Pralatrexate and Sofosbuvir) show coordination number in the range between 400 and 900, indicating many contacts formed between RdRp and the drugs. (ii), lowest free energy for the Azithromycin and Pralatrexate without curve shift. (iv) lowest free energy for the Amoxicillin and Sofosbuvir without curve shift. B(i) and (ii) The interaction patterns between Amoxicillin and RdRp, Sofosbuvir with RdRp, respectively. Last frame of MD simulation trajectories were used. Their interaction patterns have deviated from the initial docking conformation according to large RMSD observed in S2B Fig (iii) and (iv), the representative conformations corresponding to lowest energy basin of RdRp-Azithromycin and RdRp-Pralatrexate from metadyanmcis simulations. (TIF) [file pcbi.1008489.s004.tif]

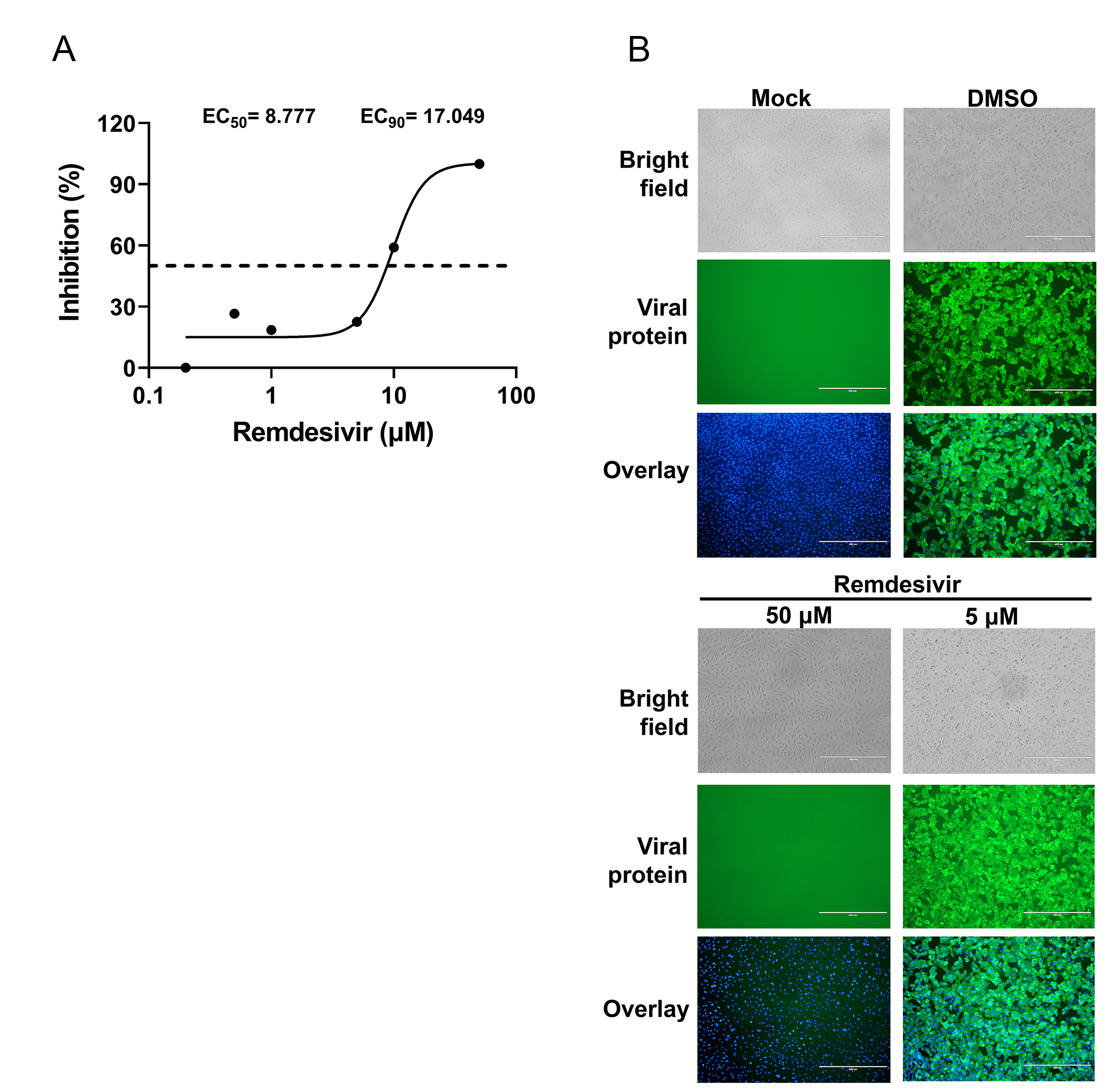

Supplement: S5 Fig — A, Vero cells were infected with SARS-COV-2 at an MOI of 0.02 in the presence of the indicated concentrations of Remdesivir for 48 hours. The viral yield in the cell supernatant was then quantified by qRT-PCR. B, Immunofluorescence microscopy of virus infection upon treatment of Remdesivir at the indicated concentrations. IFA was performed at 48 hours post infection. Scale bar, 100 μm. Cells were immunostained for the Viral protein (green) and DNA (blue). (TIF) [file pcbi.1008489.s005.tif]

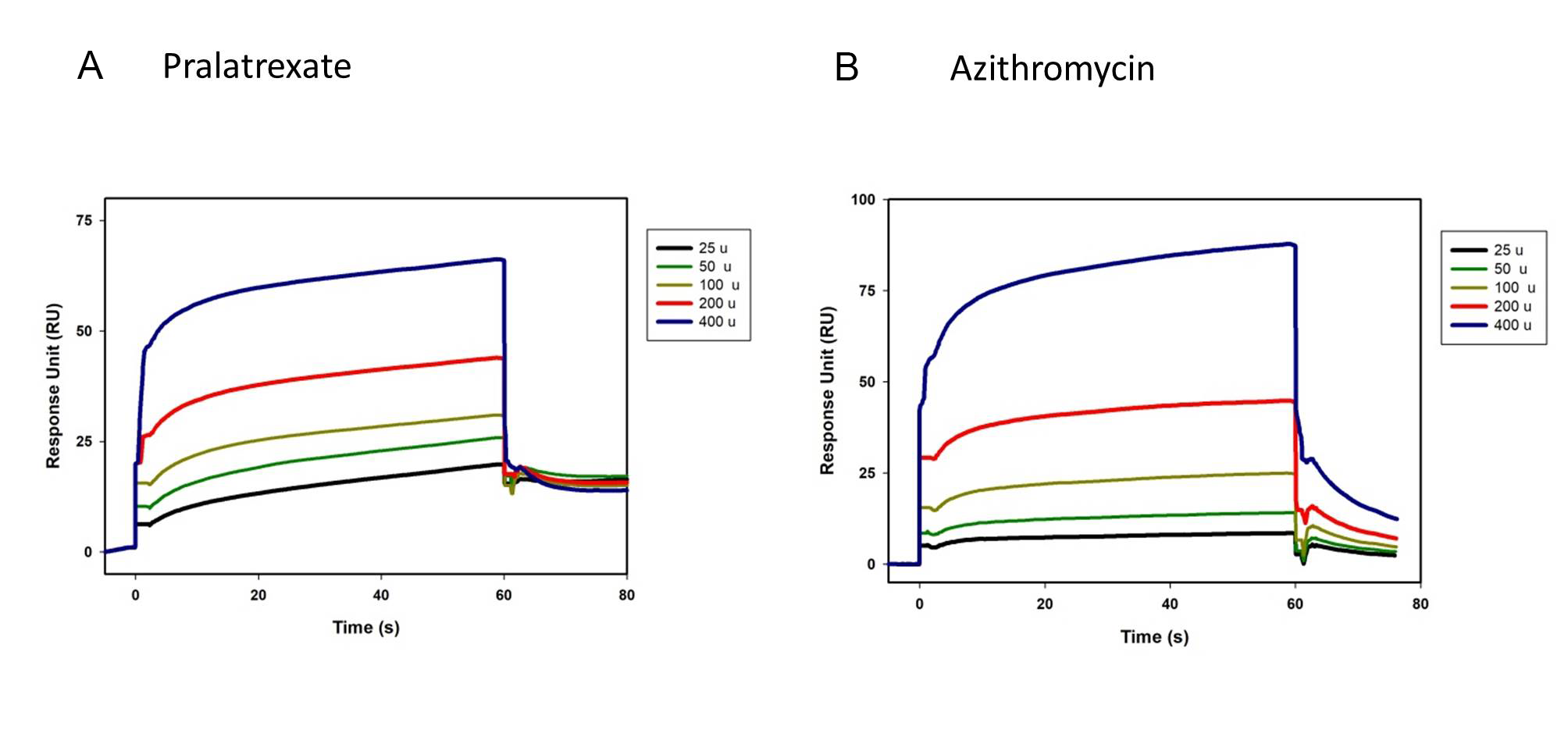

Supplement: S6 Fig — SARS-CoV-2 nsp12 polymerase protein was immobilized on the chip and tested for binding with gradient concentrations of candidate compounds. The binding profiles of different drugs are shown in individual panels. Pralatrexate (A); Azithromycin (B). The raw binding curves are shown in the figure. The data shown is a representative result of two independent experiments using different protein preparations. (TIF) [file pcbi.1008489.s006.tif]

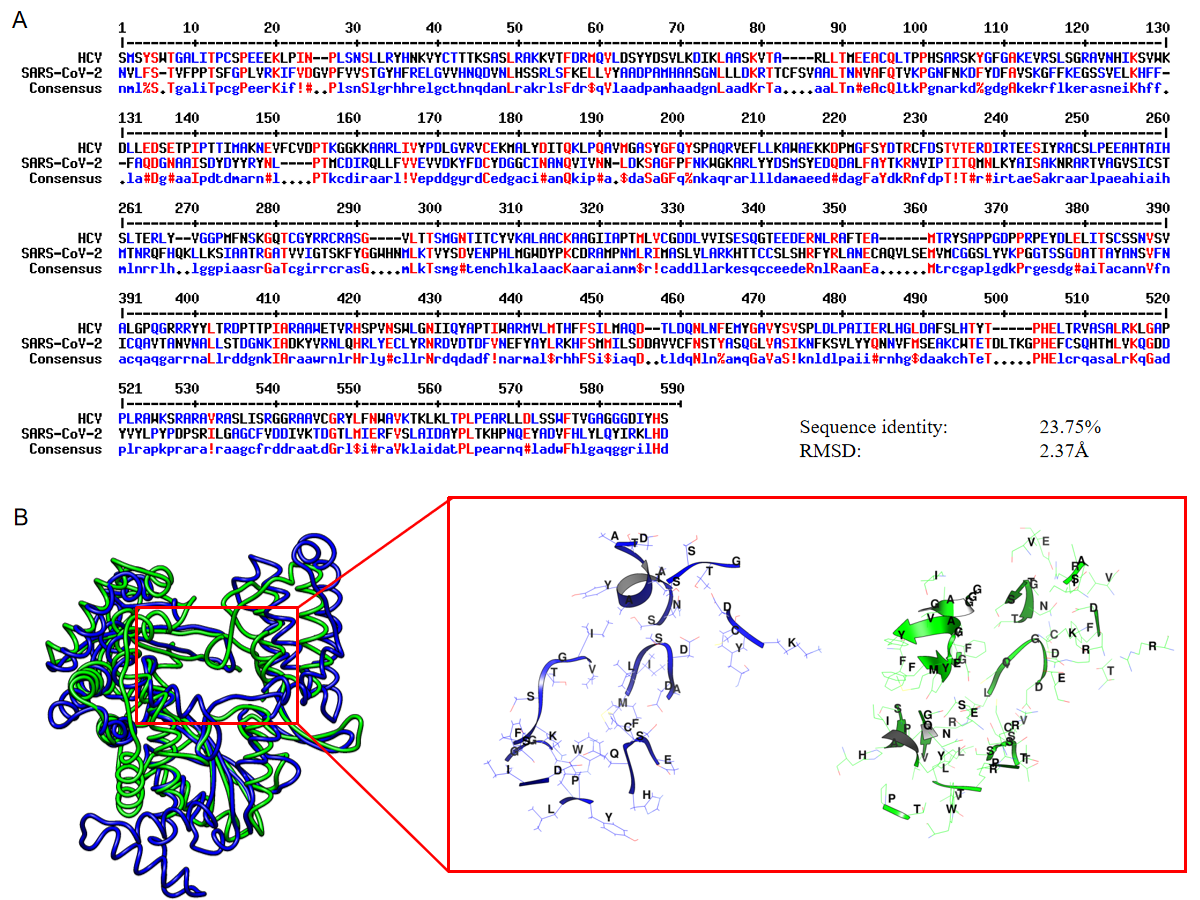

Supplement: S7 Fig — A, Sequence alignment of RdRp of HCV and RdRp of SARS-CoV-2. The low sequence identity (23.75%) between RdRp of HCV and SARS-CoV-2 may explain why Sofosbuvir inhibitor HCV but not RdRp. B, Structural superposition of RdRp 3D structures of HCV and SARS-CoV-2 (Left) shows the ligand binding region between RdRp of HCV and SARS-CoV-2 have very different residue composition. For instance there are 5 vs 3 ASPs, 2 vs 1 LYS, 1 vs 3 GLUs, 0 vs 6 ARGs in RdRp pocket of SARS-CoV-2 and RdRp pocket of HCV, respectively (Right box). Compared with the pocket of RdRp of HCV, the RdRp pocket of SARS-CoV-2 is more negatively charged. (TIF) [file pcbi.1008489.s007.tif]

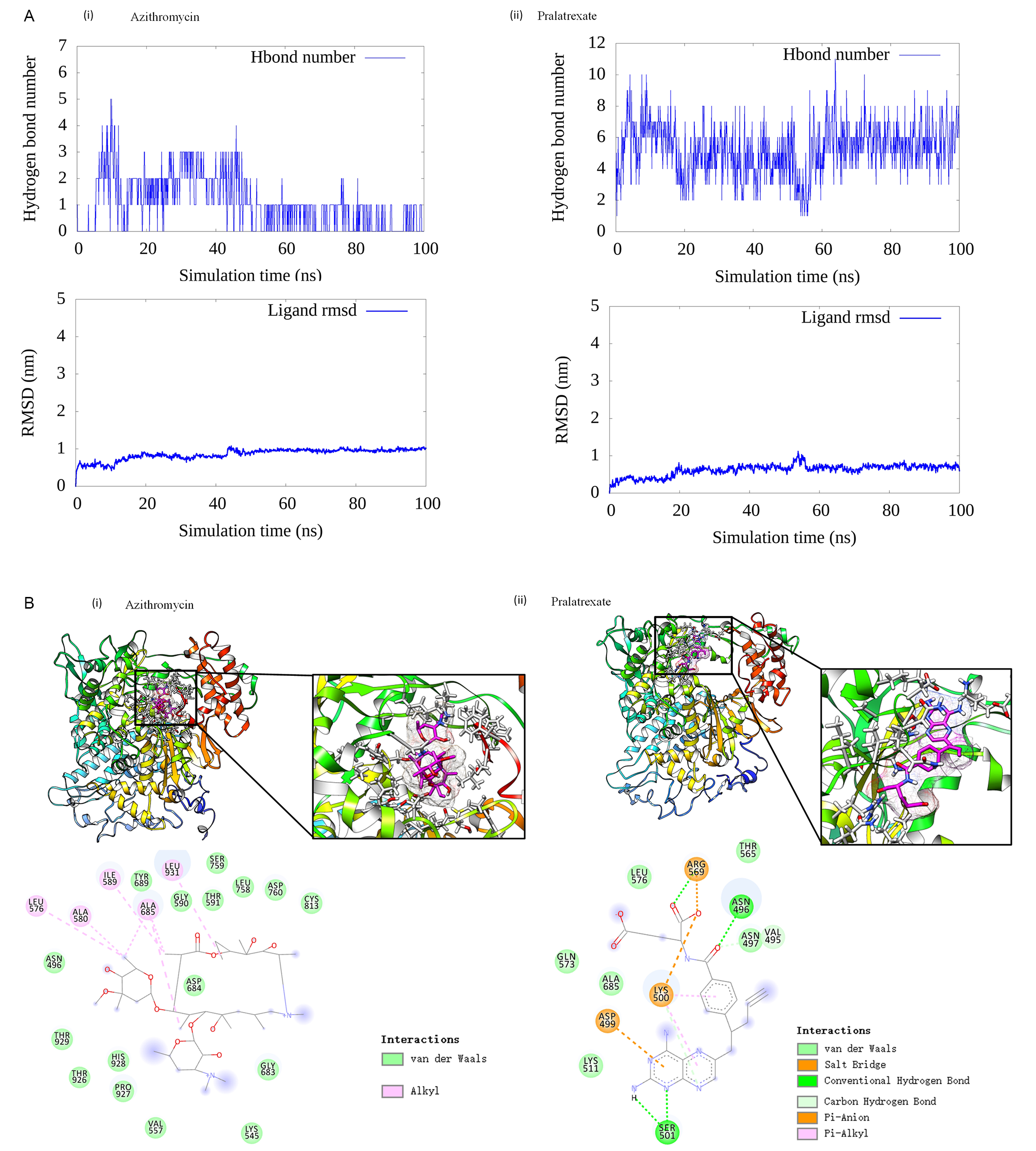

Supplement: S8 Fig — A, shows the hydrogen bond number and RMSD of ligand along the MD simulations of RdRp-Azithromycin and RdRp-Pralatrexate, respectively ((i) Azithromycin, (ii) Pralatrexate). B, the snapshot and interaction mode for RdRp-drugs from 100ns MD simulation ((i) Azithromycin, (ii) Pralatrexate). Drugs are shown as magenta stick. (TIF) [file pcbi.1008489.s008.tif]

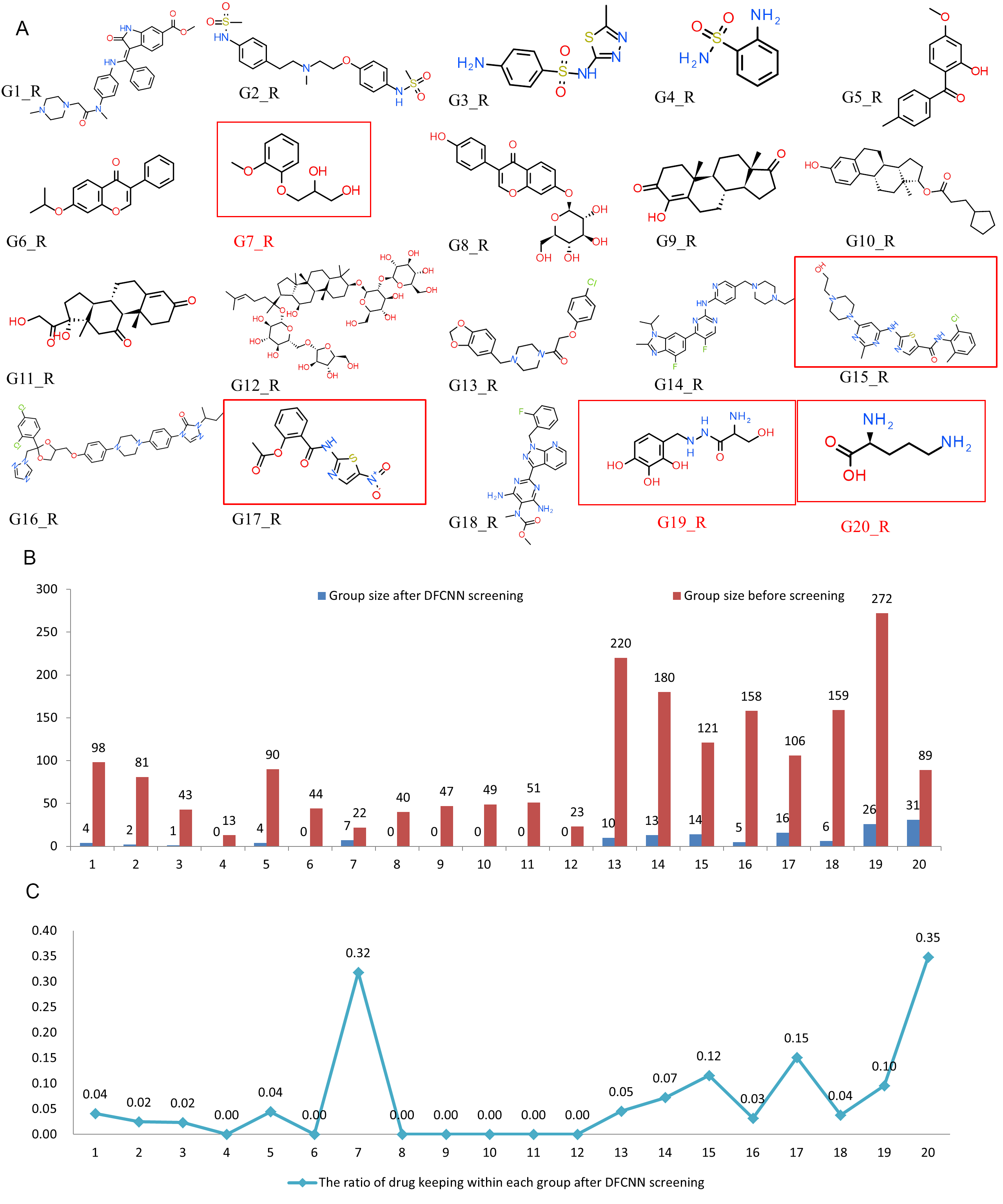

Supplement: S9 Fig — A, The 2D structure of representative drugs for the 20 groups of 1906 size dataset. B, The number of drugs in each group (red) before molecular vector-based screening, and the number of each group left (blue) after molecular vector-based screening, G20, G19, G15, G17, G7 have the more drugs left (red in panel a). C, the ratio of drugs keeping within each group after molecular vector-based screening (DFCNN). (TIF) [file pcbi.1008489.s009.tif]

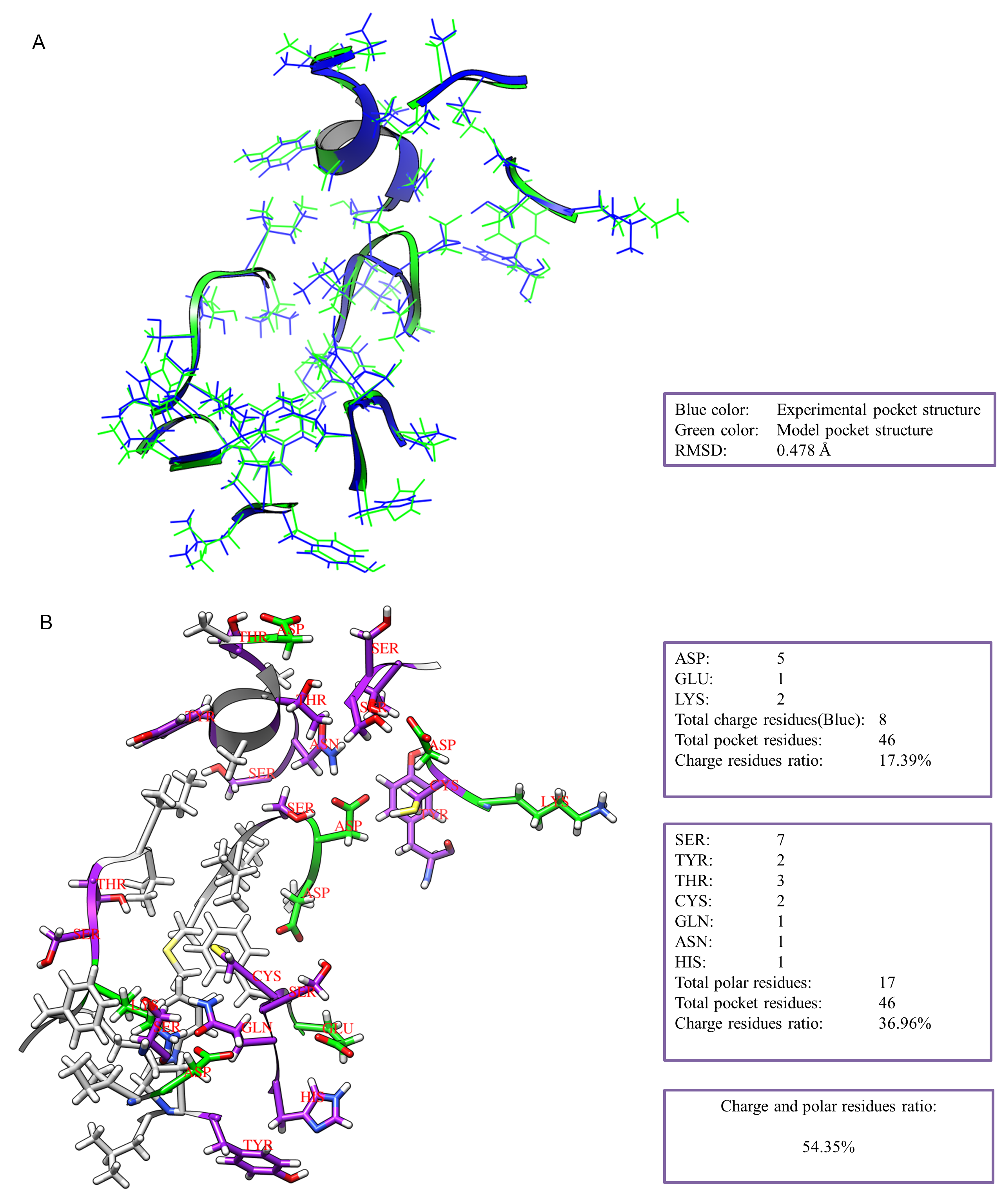

Supplement: S10 Fig — A, superposition of the modeled pocket structure with the experimental pocket structure, the two pockets are highly similar (0.478 Å). B, the residues in the modeled pocket structures. The charged residues are showed as green stick, and the polar residues are showed as purple sticks. The name labels of charge and polar residues are given with red color. (TIF) [file pcbi.1008489.s010.tif]

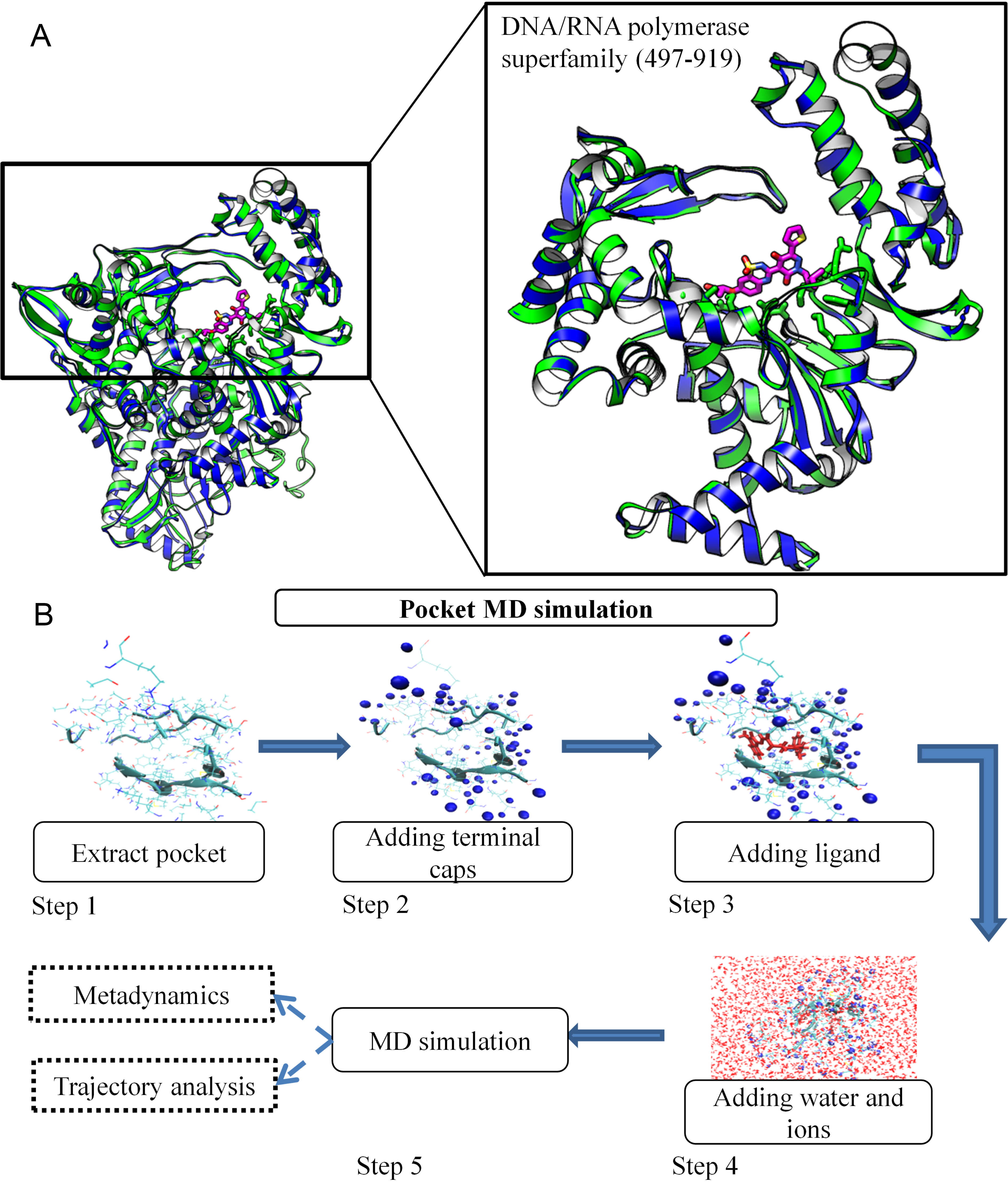

Supplement: S11 Fig — A, superimposed conformation of modeled RdRp (green) with the experimental obtained RdRp (blue), the modeled ligand are shown in magenta (Left), the DNA/RNA polymerase superfamily region, which was predicted by InterPro (https://www.ebi.ac.uk/interpro/), have high structure similarity between the modeled structure and experimental structure with RMSD of 0.456 Å (Right). B, The schematic workflow of the proposed pocket MD simulation. Step 1, Pocket extraction based on 1nm from the known ligand atoms; Step 2, Adding ACE and NHE to the N terminal and C terminal, respectively; Step 3, Adding ligand molecule topology and coordinate; Step 4, Adding water box and counter-ions; Step 5, Restrain the terminal residues, MD simulation and downstream analysis. (TIF) [file pcbi.1008489.s011.tif]

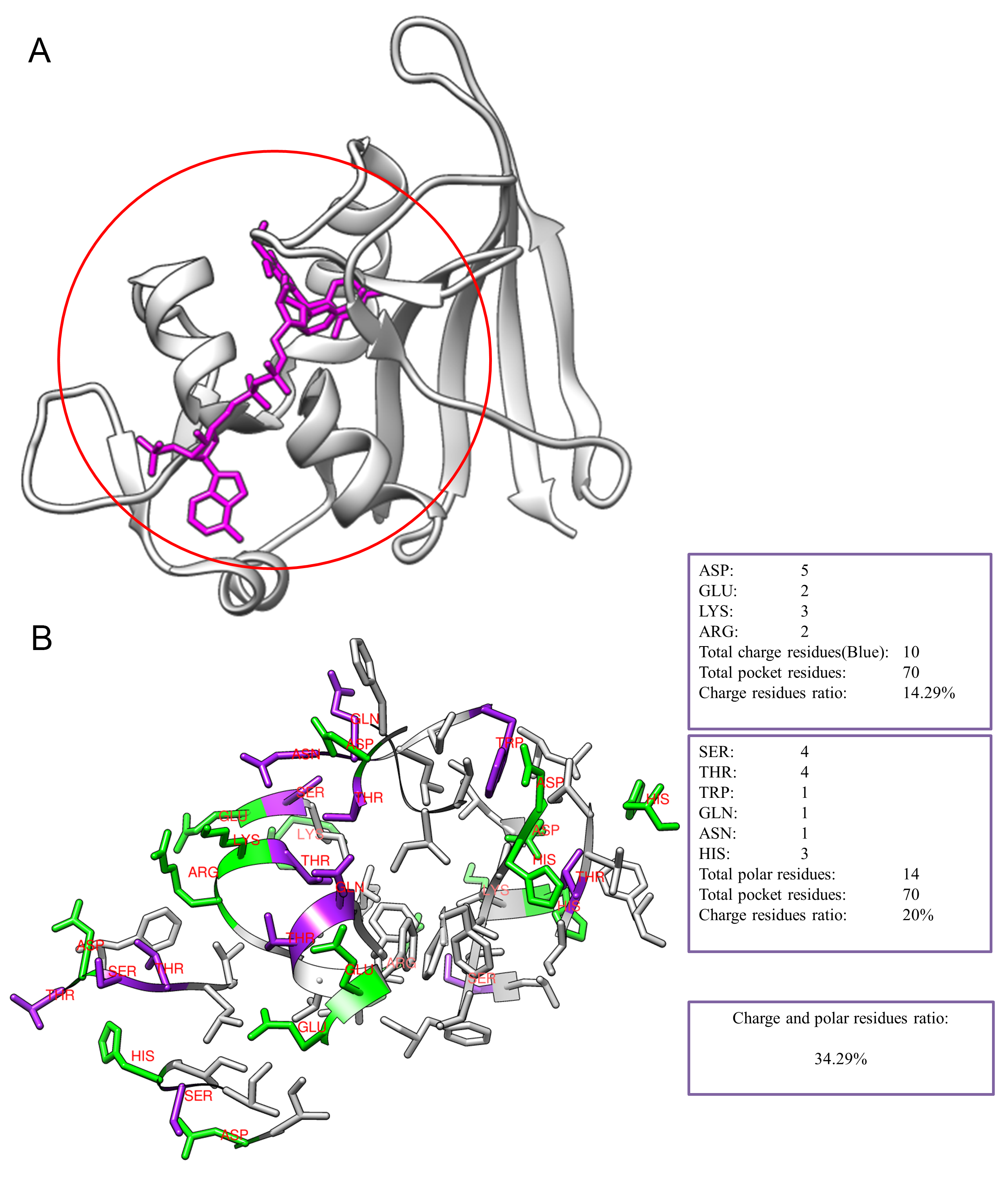

Supplement: S12 Fig — A, the DHFR protein with its two known binding ligands. B, the residues in the pocket which defined as 1 nm distance from the known ligands. The charged residues are showed as green stick, and the polar residues are showed as purple sticks. The name labels of charge and polar residues are given with red color. (TIF) [file pcbi.1008489.s012.tif]
