# Supplementary material for: A novel virtual screening procedure identifies Pralatrexate as inhibitor of SARS-CoV-2 RdRp and it reduces viral replication in vitro
Source: PLoS Comput Biol. 2020 Dec 31;16(12):e1008489. doi: 10.1371/journal.pcbi.1008489 (PMC7774833; doi:10.1371/journal.pcbi.1008489)
Supplement: S1 Table — The common residues between Azithromycin and Pralatrexate are shown in bold, the common residues between Azithromycin and Remdesivir in its monophosphate are marked with “*”. The neighbor distance criterion is 4 Å. (DOCX) [file pcbi.1008489.s013.docx]

**S1 Table.** The list of drugs that have DFCNN score above 0.9, docking score below -7 kcal/mol, and the corresponding DeepBindBC scores are presented. The drugs with DeepBindBC scores above 0.7 were indicated in bold fonts.

| Drug Name | DFCNN Score | Autodock Vina Score (kcal/mol) | DeepBindBC Score |
| --- | --- | --- | --- |
| **Amenamevir** | 0.9367 | -8.6 | 0.8810 |
| **Azithromycin** | 0.9093 | -8.6 | 0.8589 |
| **Romidepsin** | 0.9726 | -8.2 | 0.7330 |
| Ceftazidime | 0.9745 | -7.8 | 0.3272 |
| **Fipronil** | 0.9101 | -7.8 | 0.7309 |
| **Raltegravir** | 0.9845 | -7.8 | 0.7740 |
| **Amoxicillin** | 0.9844 | -7.6 | 0.9359 |
| Cephradine | 0.9447 | -7.6 | 0.3474 |
| Methotrexate | 0.9752 | -7.6 | 0.5120 |
| **Pralatrexate** | 0.9426 | -7.6 | 0.9752 |
| **Nitisinone** | 0.9923 | -7.5 | 0.7868 |
| Odanacatib | 0.9946 | -7.5 | 0.1647 |
| Quinapril | 0.907 | -7.4 | 0.2967 |
| Ampicillin | 0.9834 | -7.3 | 0.4785 |
| **Sofosbuvir** | 0.9132 | -7.3 | 0.9931 |
| **Gemcitabine** | 0.9083 | -7.2 | 0.9894 |
| **Teriflunomide** | 0.984 | -7.2 | 0.8937 |
| Cariprazine | 0.9091 | -7.1 | 0.6097 |
| **Clofarabine** | 0.9387 | -7.1 | 0.9901 |
| **Adenosine** | 0.9291 | -7 | 0.7768 |
| **Vidarabine** | 0.9291 | -7 | 0.8859 |
| Vildagliptin | 0.9665 | -7 | 0.1293 |
