# Supplementary material for: A novel virtual screening procedure identifies Pralatrexate as inhibitor of SARS-CoV-2 RdRp and it reduces viral replication in vitro
Source: PLoS Comput Biol. 2020 Dec 31;16(12):e1008489. doi: 10.1371/journal.pcbi.1008489 (PMC7774833; doi:10.1371/journal.pcbi.1008489)
Supplement: S2 Table — The drugs with DeepBindBC scores above 0.7 were indicated in bold fonts. (DOCX) [file pcbi.1008489.s014.docx]

**S2 Table.** The calculated free energy for four selected drugs based on S4A Fig (ii)(iv). The free energy landscapes of other 10 compounds are positive which indicates no binding. Since non-binding drugs are not our interest and hard to estimate exactly binding free energy value, we hasn’t list their calculated binding free energy value.

| **Name** | **StateA Free energy**  **(kJ/mol)** | **StateB free energy**  **(kJ/mol)** | **ΔG**  **(kJ/mol)** |
| --- | --- | --- | --- |
| Pralatrexate | -390.5 | -519.1 | -128.6 |
| Azithromycin | -744.8 | -1050.6 | -305.8 |
| Amoxicillin | -525.0 | -592.3 | -67.3 |
| Sofosbuvir | -462.6 | -552.5 | -89.9 |
| StateA: lowest free energy between coordination number (CV) of 0 to 100 for Pralatrexate, Azithromycin, Amoxicillin, and Sofosbuvir respectively.  StateB: lowest free energy between coordination number (CV) of 500 to 800, 900 to 1000, 500 to 700, and 400 to 600 for Pralatrexate, Azithromycin, Amoxicillin, and Sofosbuvir, respectively. | | | |
