# Supplementary material for: A novel virtual screening procedure identifies Pralatrexate as inhibitor of SARS-CoV-2 RdRp and it reduces viral replication in vitro
Source: PLoS Comput Biol. 2020 Dec 31;16(12):e1008489. doi: 10.1371/journal.pcbi.1008489 (PMC7774833; doi:10.1371/journal.pcbi.1008489)
Supplement: S3 Table — The free energy landscapes of other 10 compounds are positive which indicates no binding. Since non-binding drugs are not our interest and hard to estimate exactly binding free energy value, we hasn’t list their calculated binding free energy value. (DOCX) [file pcbi.1008489.s015.docx]

**S3 Table. The neighboring residues of Azithromycin, Pralatrexate, and Remdesivir in its monophosphate that shown in Fig 1B are presented.** The common residues between Azithromycin and Pralatrexate are shown in bold, the common residues between Azithromycin and Remdesivir in its monophosphate are marked with “*”. The neighbor distance criterion is 4 Å.

| **Azithromycin** | **Pralatrexate** | **Remdesivir in its monophosphate** |
| --- | --- | --- |
| **ALA580** | **ALA580** | ALA688* |
| **ALA685** | **ALA685** | ARG555 |
| ALA581 | ALA688* | ASN691 |
| **ARG569** | **ARG569** | ASP623 |
| **ASN496** | **ASN496** | ASP760 |
| **ASN497** | **ASN497** | CYS622 |
| ASP499 | ASP684 | LYS545 |
| **GLN573** | **GLN573** | SER682* |
| HIE928 | GLY590 | THR687 |
| **ILE494** | **ILE494** | VAL557 |
| **ILE589** | **ILE589** |  |
| **LEU576** | **LEU576** |  |
| LEU498 | LYS500 |  |
| **LYS577** | **LYS577** |  |
| THR686 | SER682* |  |
| VAL495 | THR565 |  |
