# Supplementary material for: A novel virtual screening procedure identifies Pralatrexate as inhibitor of SARS-CoV-2 RdRp and it reduces viral replication in vitro
Source: PLoS Comput Biol. 2020 Dec 31;16(12):e1008489. doi: 10.1371/journal.pcbi.1008489 (PMC7774833; doi:10.1371/journal.pcbi.1008489)
Supplement: S4 Table — (DOCX) [file pcbi.1008489.s016.docx]

**S4 Table.** 10 million scale drug screening of DFCNN on 8 representative protein targets from the DUD.E diverse data set.

| Target Name | PDBID | Description | Total_num | Active_num | Ratio_0.9_ |
| --- | --- | --- | --- | --- | --- |
| [HIVPR](http://dude.docking.org/targets/hivpr) | 1XL2 | Human immunodeficiency virus type 1 protease | 10,403,431 | 536 | 860.86 |
| [CP3A4](http://dude.docking.org/targets/cp3a4) | 3NXU | Cytochrome P450 3A4 | 10,403,065 | 170 | 12.50 |
| GCR | 3BQD | Glucocorticoid receptor | 10,403,153 | 258 | 4.54 |
| [HIVRT](http://dude.docking.org/targets/hivrt) | 3LAN | Human immunodeficiency virus type 1 reverse transcriptase | 10,403,233 | 338 | 1.84 |
| [KIF11](http://dude.docking.org/targets/kif11) | 3CJO | Kinesin-like protein 1 | 10,403,011 | 116 | 1.45 |
| [AMPC](http://dude.docking.org/targets/ampc) | 1L2S | Beta-lactamase | 10,402,943 | 48 | 1.45 |
| [AKT1](http://dude.docking.org/targets/akt1) | 3CQW | Serine/threonine-protein kinase AKT | 10,403,188 | 293 | 1.18 |
| [CXCR4](http://dude.docking.org/targets/cxcr4)^#^ | 3ODU | C-X-C chemokine receptor type 4 | 10,402,935 | 40 | 0.60 |
| *: Active_num: The number of active compounds from DUD.E dataset for a given protein target.  Total_num: The number of 10,402, 895 drug-like compounds from ZINC database plus the Active_num  Ratio_0.9_: The ratio between predicted TPR and random selection TPR. The details are described in the Materials and Methods section.  ^#^: Membrane protein | | | | | |
