# Supplementary material for: A novel virtual screening procedure identifies Pralatrexate as inhibitor of SARS-CoV-2 RdRp and it reduces viral replication in vitro
Source: PLoS Comput Biol. 2020 Dec 31;16(12):e1008489. doi: 10.1371/journal.pcbi.1008489 (PMC7774833; doi:10.1371/journal.pcbi.1008489)
Supplement: S5 Table — (DOCX) [file pcbi.1008489.s017.docx]

**S5 Table.** Number of drugs selected after Autodock Vina and DeepBindBC based screening for each Group in S9A Fig.

| Group ID | Name | Number |
| --- | --- | --- |
| 1 | Amenamevir | 1 |
| 3 | Fipronil | 1 |
| 15 | Pralatrexate, Sofosbuvir, Gemcitabine, Clofarabine, Adenosine, Vidarabine | 6 |
| 17 | Amoxicillin, Nitisinone | 2 |
| 18 | Raltegravir, Teriflunomide | 2 |
| 19 | Azithromycin, Romidepsin | 2 |
