# Supplementary material for: A novel virtual screening procedure identifies Pralatrexate as inhibitor of SARS-CoV-2 RdRp and it reduces viral replication in vitro
Source: PLoS Comput Biol. 2020 Dec 31;16(12):e1008489. doi: 10.1371/journal.pcbi.1008489 (PMC7774833; doi:10.1371/journal.pcbi.1008489)
Supplement: Attachment — Submitted filename: PCOMPBIOL-D-20-01496_R1_reviewer_with_edits.pdf [file pcbi.1008489.s019.pdf]

PLOS Computational Biology

A Novel Virtual Screening Procedure Identifies Pralatrexate as Inhibitor of SARS-CoV-2 RdRp and It Reduces Viral Replication in vitro

--Manuscript Draft--

**Manuscript Number:**	PCOMPBIOL-D-20-01496R1
**Full Title:**	A Novel Virtual Screening Procedure Identifies Pralatrexate as Inhibitor of SARS-CoV-2 RdRp and It Reduces Viral Replication in vitro
**Short Title:**	Pralatrexate and Azithromycin identified by virtual screening inhibit SARS-CoV-2 in vitro
**Article Type:**	Research Article
**Keywords:**	SARS-CoV-2; RdRp; virtual screening; Pralatrexate; Azithromycin
**Abstract:**	The spread of severe acute respiratory syndrome coronavirus 2 (SARS-CoV-2) virus poses serious threats to the global public health and leads to worldwide crisis. No effective drug or vaccine is readily available. The viral RNA-dependent RNA polymerase (RdRp) is a promising therapeutic target. A hybrid drug screening procedure was proposed and applied to identify potential drug candidates targeting RdRp from 1906 approved drugs. Among the four selected market available drug candidates, Pralatrexate and Azithromycin were confirmed to effectively inhibit SARS-CoV-2 replication in vitro with EC_{50} values of 0.008μM and 9.453 μM, respectively. For the first time, our study discovered that Pralatrexate is able to potently inhibit SARS-CoV-2 replication with a stronger inhibitory activity than Remdesivir within the same experimental conditions. The paper demonstrates the feasibility of fast and accurate anti-viral drug screening for inhibitors of SARS-CoV-2 and provides potential therapeutic agents against COVID-19.
**Additional Information:**	
**Question**	**Response**
**Financial Disclosure**<br><br>Enter a financial disclosure statement that describes the sources of funding for the work included in this submission. Review the submission guidelines for detailed requirements. View published research articles from *PLOS Computational Biology* for specific examples.<br><br>This statement is required for submission and **will appear in the published article** if the submission is accepted. Please make sure it is accurate.	This work was partly supported by Strategic Priority CAS Project XDB38000000, the National Key Research and Development Program of China under Grant No. 2018YFB0204403, 2016YFB0201305 and 2019YFA0906100, National Science and Technology Major Project under Grant No. 2018ZX10101004, Shenzhen Basic Research Plan under Grant No. JCYJ20190807170801656, National Science Foundation of China under Grant no. U1813203 and 61433012; the National Natural Youth Science Foundation of China (Grant no. 31601028), the Shenzhen Basic Research Fund under Grant no. JCYJ20180507182818013, JCYJ20170413093358429 and GGFW2017073114031767, the China Postdoctoral Science Foundation under Grant no. 2019M653132, CAS Key Lab under Grant no. 2011DP173015, and the SIAT Innovation Program for Excellent Young Researchers. We would also like to thank the funding support by the Shenzhen Discipline Construction Project for Urban Computing and Data Intelligence, Youth Innovation Promotion Association, CAS to Yanjie Wei.

Powered by Editorial Manager® and ProduXion Manager® from Aries Systems Corporation

**Unfunded studies**<br>Enter: *The author(s) received no specific funding for this work.*<br><br>**Funded studies**<br>Enter a statement with the following details:<br>• Initials of the authors who received each award<br>• Grant numbers awarded to each author<br>• The full name of each funder<br>• URL of each funder website<br>• Did the sponsors or funders play any role in the study design, data collection and analysis, decision to publish, or preparation of the manuscript?<br>• **NO** - Include this sentence at the end of your statement: *The funders had no role in study design, data collection and analysis, decision to publish, or preparation of the manuscript.*<br>• **YES** - Specify the role(s) played.<br><br>* typeset	
**Competing Interests**<br><br>Use the instructions below to enter a competing interest statement for this submission. On behalf of all authors, disclose any competing interests that could be perceived to bias this work—acknowledging all financial support and any other relevant financial or non-financial competing interests.<br><br>This statement **will appear in the published article** if the submission is accepted. Please make sure it is accurate. View published research articles from *PLOS Computational Biology* for specific examples.	NO authors have competing interests

**NO authors have competing interests**<br><br>Enter: *The authors have declared that no competing interests exist.*<br><br>**Authors with competing interests**<br><br>Enter competing interest details beginning with this statement:<br><br>*I have read the journal's policy and the authors of this manuscript have the following competing interests: [insert competing interests here]*<br><br>* typeset	
**Data Availability**<br><br>Authors are required to make all data underlying the findings described fully available, without restriction, and from the time of publication. PLOS allows rare exceptions to address legal and ethical concerns. See the PLOS Data Policy and FAQ for detailed information.<br><br>A Data Availability Statement describing where the data can be found is required at submission. Your answers to this question constitute the Data Availability Statement and **will be published in the article**, if accepted.<br><br>**Important:** Stating 'data available on request from the author' is not sufficient. If your data are only available upon request, select 'No' for the first question and explain your exceptional situation in the text box.<br><br>Do the authors confirm that all data underlying the findings described in their manuscript are fully available without restriction?	Yes - all data are fully available without restriction
**Describe where the data may be found in full sentences. If you are copying our sample text, replace any instances of XXX with the appropriate details.**	All relevant data are within the manuscript and its Supporting Information files.

• If the data are **held or will be held in a public repository**, include URLs, accession numbers or DOIs. If this information will only be available after acceptance, indicate this by ticking the box below. For example: *All XXX files are available from the XXX database (accession number(s) XXX, XXX.)*.<br>• If the data are all contained **within the manuscript and/or Supporting Information files**, enter the following: *All relevant data are within the manuscript and its Supporting Information files.*<br>• If neither of these applies but you are able to provide **details of access elsewhere**, with or without limitations, please do so. For example:<br><br>*Data cannot be shared publicly because of [XXX]. Data are available from the XXX Institutional Data Access / Ethics Committee (contact via XXX) for researchers who meet the criteria for access to confidential data.*<br><br>*The data underlying the results presented in the study are available from (include the name of the third party and contact information or URL).*<br>• This text is appropriate if the data are owned by a third party and authors do not have permission to share the data.<br><br>* typeset	
Additional data availability information:	Tick here if the URLs/accession numbers/DOIs will be available only after acceptance of the manuscript for publication so that we can ensure their inclusion before publication.

Powered by Editorial Manager® and ProduXion Manager® from Aries Systems Corporation

Reviewer: 1

Comments

The authors report an interesting study involving various *in silico* approaches for drug repositioning and experimental validation of some selected compounds. In my opinion some sentences need some additional explanations. My points are reported below:

Preparation of TargetMol-Approved_Drug_Library in 3D for docking?

Protonation state? 3D structure generator..? no warning / flag alerts for highly toxic compounds ?

When docking with Autodock Vina, the authors keep the best pose or they also investigate some top poses for each ligand as, the best energy pose does not mean that this is true pose in the binding pocket

I do not understand this sentence in the method: clustering 1906 drugs into 20 groups? There should be much more than 20 groups? or it depends how the groups are defined.

Could it be that Azithromycin has some good docking score only because it is big?

Could it be that drugs that are small or making less contacts have high RMSD fluctuations ?and if so, the type of simulation used mainly confirm that a large compound with many polar interactions will stick to a protein while a small compound will be unstable… If so, the tool does not discriminate anything? Please clarify or comment

I do not understand these sentences:

starting line: 177:

The possible explanation is that our protein-drug systems do not contain the RNA primers during the MD simulation, and covalent bond formation, such as the Remdesivir in its monophosphate form, could not be estimated by traditional MD simulation.

Few lines after, if some of the drugs used here are covalent binders, this should be explained further, please optimize the sentence

The authors suggest that Pralatrexate is already known to be active on Cov2 (as seen in the GHDDI list) but that in that list, the molecular mechanism of action of Pralatrexate has never been described or never shown to involve the RdRp target? Is it correct? and thus that Pralatrexate not only inhibits DHFR but also RdRp? If so, is there some similarity between the binding site of both targets? Please comment

>>Preparation of TargetMol-Approved_Drug_Library in 3D for docking? Protonation state ? 3D structure generator..? no warning / flag alerts for highly toxic compounds ?

Response: These are very good points. The TargetMol-Approved_Drug_Library is well prepared by the TargetMol Company in 3D with sdf format. We have prepared the docking ligand structure using standard procedure used in many drug discovery projects. Briefly, the steps are as follows. The ligand was converted from sdf to mol2 by Openbabel, and then converted into pdbqt by scripts in AutoDockTools. Since the Autodock Vina only considers the heavy atoms, and the polar hydrogens, the

protonation state before docking is determined by the default methods in AutoDockTools. It should be noted that before MD simulation, we carefully visualized and checked each docked ligand manually to make sure they are in correct protonation state by pymol software [1]. At this first stage, we haven't considered whether a compound is highly toxic or not because the most of compounds in this dataset are FDA approved drugs, having passed human toxicity tests prior to approval. We hence assumed they should be clinically safe for human uses. We have added this detailed information in Line 471-477 and Line 491-493 of the revised manuscript.

>>When docking with Autodock Vina, the authors keep the best pose or they also investigate some top poses for each ligand as, the best energy pose does not mean that this is true pose in the binding pocket

Response: Thanks for your helpful advice. Exactly, we considered this when using DeepBindBC, so we estimated and analyzed each of the generated docking poses, and then choose the top score as the final score. Here, we used the best Autodock Vina score corresponding pose as initial conformation for the next stage MD simulation. The best docking score pose is not guaranteed to be always correct as you suggested. However, the candidates of interest were subjected to MD simulation later, and MD simulation itself may be able to sample the correct binding pose if the initial structure dose not deviated too large. Usually, the last frame of MD simulation or the representative conformation of the largest cluster is more likely to be the correct binding pose than Docking.
>>I do not understand this sentence in the method: clustering 1906 drugs into 20 groups ? There should be much more than 20 groups? or it depends how the groups are defined.

Response: Thanks for your helpful advice. Many clustering methods require an initial arbitrary definition of number of clusters or cutoff values. In this study, we used the algorithm of Murtagh[2] by Clusfps (https://github.com/kaiwang0112006/clusfps) to do the clustering. This method requires the users to define the group number. We have tried different value of group number, and find 20 group can generate groups member with very similar drug properties (charge, polar, size, or hydrophobic, *etc*). We also attempt to do clustering with the algorithm of Butina [3], in which no need extra cluster number definition. But it still requires to define a cutoff value, which is also some arbitrary.
>>Could it be that Azithromycin has some good docking score only because it is big?
Response: Very good point, thanks for the concerns. The Docking score function sometimes favors hydrophobic interaction, and selects compound with large size according to our previous study [4] and other literature reports[5]. That is why we usually won't use docking score as the only criterion to select drug candidates. It can be noticed that Azithromycin also has very good DFCNN and DeepBindBC scores which have no bias on hydrophobic interaction. Furthermore, Azithromycin is also stable in MD simulation， and favors binding on the pocket in metadynamics based

free energy calculation. Because several methods we used all predict Azithromycin likely to binding with RdRp pocket, we choose it for final experimental validation.

>>Could it be that drugs that are small or making less contacts have high RMSD fluctuations? and if so, the type of simulation used mainly confirm that a large compound with many polar interactions will stick to a protein while a small compound will be unstable… If so, the tool does not discriminate anything ? Please clarify or comment

Response: Thanks for your helpful advice. We have checked the RMSD fluctuations of drugs that are small or with fewer contacts as you suggested. And we found that there is no direct correlation between RMSD fluctuation and size (or number of initial contacts). For instance, the Romidepsin has very large size (molecular weight: 544.7), but shows less binding stability compared to Pralatrexate (molecular weight: 479.5). Also, Vidarabine have molecular weight of 271.3, shows more stable binding compared to Sofosbuvir (molecular weight: 527) and Amenamevir (molecular weight: 484.6). In MD simulation, small size ligands can also have very tight binding depending on the ligand and binding site (pocket) property[6]. Whether the binding is stable is more related to the complementary between pocket and ligand in both geometric and physio-chemical features. So the RMSD calculations can help to provide useful information about the binding stability. It should be noted that we use the calculated free energy to do the final decision about which compounds should be chosen for last stage experimental validation, while the ligand RMSD in the MD simulation are not so reliable for judging whether a drug is a good candidates in some cases, especially when the initial binding conformation is deviated from the native one. We have clarified it in the Line 174-183 with above sentences.

>>I do not understand these sentences:
starting line: 177:
The possible explanation is that our protein-drug systems do not contain the RNA primers during the MD simulation, and covalent bond formation, such as the Remdesivir in its monophosphate form, could not be estimated by traditional MD simulation.

Few lines after, if some of the drugs used here are covalent binders, this should be explained further, please optimize the sentence
Response: Thanks for your helpful advice. We have not used any drugs that are covalent binders. Yes, it is quite easy to mislead by using this sentence. We have changed this sentence into “The possible explanation is that our protein-drug systems do not contain the RNA primers during the MD simulation, and without RNA the nucleoside analogues would not form base pair like interaction, hence shows no binding in MD simulation.” in Line 208-210 of the revised manuscript.

>>The authors suggest that Pralatrexate is already known to be active on Cov2 (as

seen in the GHDDI list) but that in that list, the molecular mechanism of action of Pralatrexate has never been described or never shown to involve the RdRp target ? Is it correct ?and thus that Pralatrexate not only inhibits DHFR but also RdRp ? If so, is there some similarity between the binding site of both targets ? Please comment

Response: Thanks for your helpful advice. No, only the Azithromycin is already known to be active on inhibiting SARS-CoV-2 replication (as seen in the GHDDI list). But in that list, the molecular mechanism of action of Azithromycin has never been described or never shown to involve the RdRp target. For the Pralatrexte, our study is the first time to find its potent inhibition on SARS-CoV-2 replication. Pralatrexate is known to inhibit DHFR before and our study indicates it also inhibits RdRp to reduce the SARS-CoV-2 replication.

Thanks for the helpful advice about checking the binding site similarity between DHFR and RdRp. Interestingly, we found some similarities after careful investigation, for instance both the ligand binding pockets of DHFR and RdRp contain many charged and polar residues, and have similar number of different charged residues, *e.g.* both contains 5 ASP residues. And we added a paragraph to describe this in Line 390-398, and added a Supplementary Figure 12.

Reviewer: 2

Comments

The manuscript by Zhang et al. presents very interesting results obtained by applying different in silico methods, followed by reliable experimental validation. Methods are fine and results are well presented. However, some stylistic improvements are needed:

1. In the title: the viral titer is defined as the lowest concentration of virus (number of infectious units / ml) that can still infects cells. Lowering the viral titer actually means "making the virus more virulent", since a lower virus concentration is needed to infect the cell. I suggest changing in "Reduces Viral Replication" or something similar.
2. Lines 69-70: the authors states that "Several computational drug screening methods..." but only cite their own work. Please include some other citation (from a quick googling: https://doi.org/10.1016/j.jiph.2020.06.016; https://doi.org/10.1021/acs.jproteome.0c00383; https://www.nature.com/articles/s41598-020-70863-9; https://doi.org/10.1002/minf.202000115)
3. Fig. 1: please edit figures to make text readable (I'm no more in my 20th, but reading was hard even when wearing glasses...)
4. Lines 118-121: the paragraph (from "Molecular" to "top hits") needs to be rephrased for better clarity.
5. Line 123: "rejected"..."that had poor..."
6. Lines 168-169: van der Waals
7. Line 170: "metadynamics"
8. Lines 173-174: energies in kJ/mol should be rounded to one decimal place

9: Lines 192-194: the paragraph (from "A recent" to "Table 1") needs to be rephrased for better clarity.
10. Line 200: "interacting....which is consist of"
11. Line 202: "it is possible that Azithromycin and Pralatrexate occupy"
12. Line 208: "activity of Azithromycin,..."
13. Line 229: I would not say that the results is "obvious" (maybe "expected")
14. Lines 275-276 "its extremely low EC50"
15. Line 334: "many electron donors and acceptors"
16. Line 367: "within I-TASSER"
17. Lines 394-404: do the reported parameters belong to the current work or are them from ref 11? Please only report relevant information
18. Line 414: "PDB"
19. Line 439: "Note that the..."
20. Lines 445-446: from "Terminals will" to "pocket" needs rephrasing21: Line 451: "ions were added"
22. Lines 459-460: check spacing
23. Lines 467-468: "indicates that protein-lingand..."
24. Line 474: which "function"?
25. Line 574: "Thanks to TargetMol for providing"
26. Line 829. "The physico-chemical"

>>1. In the title: the viral titer is defined as the lowest concentration of virus (number of infectious units / ml) that can still infects cells. Lowering the viral titer actually means "making the virus more virulent", since a lower virus concentration is needed to infect the cell. I suggest changing in "Reduces Viral Replication" or something similar.

Response: Thanks for the good comments. Yes, previous title will lead to misunderstanding. We have revised the title into “A Novel Virtual Screening Procedure Identifies Pralatrexate as Inhibitor of SARS-CoV-2 RdRp and It Reduces Viral Replication in vitro” in the revised manuscript according to your helpful suggestion.

>>2. Lines 69-70: the authors states that "Several computational drug screening methods..." but only cite their own work. Please include some other citation (from a quick googling: https://doi.org/10.1016/j.jiph.2020.06.016; https://doi.org/10.1021/acs.jproteome.0c00383; https://www.nature.com/articles/s41598-020-70863-9; https://doi.org/10.1002/minf.202000115)

Response: Thanks for the helpful suggestion. As per your suggestion, we have added above relevant citations of methods about computational drug screening in Line 82-84 .

>>3. Fig. 1: please edit figures to make text readable (I'm no more in my 20th, but reading was hard even when wearing glasses...)

Response: Thank you. It is a very helpful suggestion. We have changed fonts size in the Figure 1, and make it more clear and readable in the revised manuscript.

>>4. Lines 118-121: the paragraph (from "Molecular" to "top hits") needs to be rephrased for better clarity.

Response: Thanks for the comment. We have carefully checked the writing in those Lines, and revised this paragraph for better clarity. For instance, previous sentence “Molecular vector-based deep learning method (DFCNN), structure feature-based deep learning method (DeepBindBC) and molecular docking (Autodock Vina) are complementary, which can be seen from Supplementary Table 2 that three methods predict different drug compounds as top hits.” is changed into “Interestingly, from Supplementary Table 2, we notice that DFCNN, DeepBindBC and Autodock Vina predict different drug compounds as top hits. This indicates that these three methods may potentially complementary to each other through judging protein-ligand interactions from different perspectives” in the Line 131-134.

>> 5. Line 123: "rejected"..."that had poor..."
6. Lines 168-169: van der Waals
7. Line 170: "metadynamics"
8. Lines 173-174: energies in kJ/mol should be rounded to one decimal place9: Lines 192-194: the paragraph (from "A recent" to "Table 1") needs to be rephrased for better clarity.
10. Line 200: "interacting....which is consist of"
11. Line 202: "it is possible that Azithromycin and Pralatrexate occupy"
12. Line 208: "activity of Azithromycin,..."
13. Line 229: I would not say that the results is "obvious" (maybe "expected")
14. Lines 275-276 "its extremely low EC50"
15. Line 334: "many electron donors and acceptors"
16. Line 367: "within I-TASSER"
17. Lines 394-404: do the reported parameters belong to the current work or are them from ref 11? Please only report relevant information
18. Line 414: "PDB"
19. Line 439: "Note that the..."
20. Lines 445-446: from "Terminals will" to "pocket" needs rephrasing
21: Line 451: "ions were added"
22. Lines 459-460: check spacing
23. Lines 467-468: "indicates that protein-lingand..."
24. Line 474: which "function"?
25. Line 574: "Thanks to TargetMol for providing"

26. Line 829. "The physico-chemical"
Response: Thanks for the helpful advices. We have corrected all above mentioned problems in the revised manuscript.
For part two of Question 8, we have changed the sentence into “In a recent study, the authors have shown that the monophosphate active form of Remdesivir interacts with the RdRp and covalently incorporated into partial double stranded RNA template of RdRp at the +1 position [7].” In Line 225-227. For Question 17, the reported results from DFCNN are using the same parameters as the current work. We have clarified this in the revised manuscript in Line 360.

Reviewer #3

Comments

1/ In the Force field based simulations 14 compounds are processed. However the free energy of only 4 compounds is given in supp table 3. Why are the other 10 compounds not listed?
2/ It would also be interesting to calculate the free binding energy of Remdesivir because it is also tested in vitro.
3/ Why are only those 4 compounds tested in vitro? what is the rationale to select only those ones?
4/ The experimental activities of the 2 compounds mentioned in the abstract do not correlate with the free energy of binding in supp table 3. Any explanation?
5/ check English + typo's: metadyanmics, ...
6/ DATA AVAILABILITY: Do not write "structure has been deposited in the PDB" but "structure has been retrieved ...". Those structures are the work of other research groups.
>>Question 1/ In the Force field based simulations 14 compounds are processed. However the free energy of only 4 compounds is given in supp table 3. Why are the other 10 compounds not listed?
Response: Thanks for the helpful question. The free energy landscapes of other 10 compounds are shown in Supplementary Fig. 4a(i). It shows that they are much favor in water than binding in the pocket. In such case, the binding free energy would be positive and we can’t accurately estimate the free energy value; it clearly indicates the ligands not prefer to binding. We have added a sentence in the Supplementary Table 3 of the revised manuscript to clarify this in the revised manuscript.

>>2/ It would also be interesting to calculate the free binding energy of Remdesivir because it is also tested in vitro.
Response: Thanks for the valuable suggestions. However, we find Remdesivir has covalent bind with the RNA primer, our method can only estimate binding free energy between non-colvalent-binding protein and ligand. Furthermore, our system not contains RNA premier, so the interaction mechanism and pattern of the Pralatrexate and Azithromycin would be different from Remdesivir. We have added more discussion about this in Line 201-205 of the revised manuscript, to make this clear.

>>3/ Why are only those 4 compounds tested in vitro? what is the rationale to select only those ones?
Response: Thanks for the helpful question. In-vitro large scale testing of compounds is labour intensive and expensive. We select those 4 compounds based on our screening pipeline. As shown in Figure 1a, we first use DFCNN, autodock Vina and DeepBindBC to preliminary screen a set of potential drugs. The using more accurate while computational expensive Molecular dynamics simulation and metadynamics simulation to further screening highly potential drug binders of RdRp. We assume the free energy calculated based on metadynamics is more reliable than other methods, although it requires relatively more computational resources. So we put this step in a last stage of screening, and finally we find only those 4 compounds have negative calculated free energy (indicate favor over binding in the pocket). That’s why we only chosen those 4 compounds for experimental validation.
>>4/ The experimental activities of the 2 compounds mentioned in the abstract do not correlate with the free energy of binding in supp table 3. Any explanation?
Response: Thanks for the helpful question. Yes, we also noticed this. The computational methods of free energy calculations include several limitations. This includes approximations involved for deriving force field parameters, and approximations in estimating energy contributions. An exact correlation between computational calculation and experimental free energy is not possible. Nonetheless, our computational calculations show good approximations when used in a screening context. We listed detailed possible reason in follow:

1. The free energy calculated by Metadynamics simulation is highly dependent on the CV we chosen, so with only one CV (coordinate number), it is possible that calculated free energy is not so accurate.
2. The MD simulation parameter currently used is still having large space to improve. So the exact binding affinity should be determined by experimental method.
3. The experimental activities may not always directly correlate with binding free energy, for instance, weak binding on critical residues may demonstrate better activity than binding on non-critical residues.

So it is quite complicated for some cases in estimating exact compound activity by purely metadynamics based free energy calculation. We have added an extra discussion about this in Line 297-305 of the revised manuscript.

>>5/ check English + typo's: metadyanmics, ...
Response: Thanks for the helpful advice. We have changed this typing error in the modified manuscript.

>>6/ DATA AVAILABILITY: Do not write "structure has been deposited in the PDB" but "structure has been retrieved ...". Those structures are the work of other research groups.
Response: Thanks for your helpful comments. We have changed the writing into “The structures for SARS-CoV-2 RdRp have been retrieved from the Protein Data Bank

with accession ID 6M71, 6WTT. The template structure we used to construct RdRp-ligand complex is retrieved from the Protein Data Bank with accession ID 3BR9).”

Problem about the data availability:

Have all data underlying the figures and results presented in the manuscript been provided?

We have deposited all data underlying the figures and results into the github (https://github.com/haiping1010/SARS_RdRp_drug_screening_data) for the convenience of readers. The MD trajectory and some original experimental data which were too large to deposit in github were putted in figshare.com with a download link (https://figshare.com/s/c63312c68952cdf6b586). We added this information in the revised manuscript.

Revised Manuscript (clean copy) Click here to access/download;Manuscript;manuscript-without-mark.docx

A Novel Virtual Screening Procedure Identifies Pralatrexate as Inhibitor of SARS-CoV-2 RdRp and It Reduces Viral Replication *in vitro*

Haiping Zhang[1]*, Yang Yang[2]*, Junxin Li[3]*, Min Wang[4], Konda Mani Saravanan[1], Jinli Wei[2], Justin Tze-Yang Ng[5], Md. Tofazzal Hossain[1,6], Maoxuan Liu[3], Huiling Zhang[1], Xiaohu Ren[7], Yi Pan[8], Yin Peng[9], Yi Shi[4], Xiaochun Wan[3†], Yingxia Liu[2†], Yanjie Wei[1†]

[1]Center for High Performance Computing, Joint Engineering Research Center for Health Big Data Intelligent Analysis Technology, Shenzhen Institutes of Advanced Technology, Chinese Academy of Sciences, Shenzhen, Guangdong, PR China 518055

[2]Shenzhen Key Laboratory of Pathogen and Immunity, National Clinical Research Center for infectious disease, State Key Discipline of Infectious Disease, Shenzhen Third People's Hospital, Second Hospital Affiliated to Southern University of Science and Technology, Shenzhen, China

[3]Shenzhen Laboratory of Human Antibody Engineering, Institute of Biomedicine and Biotechnology, Shenzhen Institutes of Advanced Technology, Chinese Academy of Sciences, 1068 Xueyuan Boulevard, University City of Shenzhen, Xili Nanshan, Shenzhen, 518055, China

[4]CAS Key Laboratory of Pathogenic Microbiology and Immunology, Institute of Microbiology, Chinese Academy of Sciences

[5]School of Biological Sciences, Nanyang Technological University, 60 Nanyang Drive, Singapore, 637551

[6]University of Chinese Academy of Sciences, No.19(A) Yuquan Road, Shijingshan District,

Beijing, P.R.China 100049
[7]Institute of Toxicology, Shenzhen Center for Disease Control and Prevention, No 8 Longyuan Road, Nanshan District, Shenzhen, 518055, China
[8]Department of Computer Science, Georgia State University, Atlanta, 30302-5060, USA.
[9]Department of Pathology, School of Medicine, Shenzhen University, Shenzhen, Guangdong, China
* These authors contributed equally
† Corresponding to: Yanjie Wei (yj.wei@siat.ac.cn)，Xiaochun Wan (xc.wan@siat.ac.cn), Yingxia Liu (yingxialiu@hotmail.com)

Abstract

The spread of severe acute respiratory syndrome coronavirus 2 (SARS-CoV-2) virus poses serious threats to the global public health and leads to worldwide crisis. No effective drug or vaccine is readily available. The viral RNA-dependent RNA polymerase (RdRp) is a promising therapeutic target. A hybrid drug screening procedure was proposed and applied to identify potential drug candidates targeting RdRp from 1906 approved drugs. Among the four selected market available drug candidates, Pralatrexate and Azithromycin were confirmed to effectively inhibit SARS-CoV-2 replication *in vitro* with EC_{50} values of 0.008μM and 9.453 μM, respectively. For the first time, our study discovered that Pralatrexate is able to potently inhibit SARS-CoV-2 replication with a stronger inhibitory activity than Remdesivir within the same experimental conditions. The paper demonstrates the feasibility of fast and accurate anti-viral drug screening for inhibitors of SARS-CoV-2 and provides potential therapeutic agents against COVID-19.

Keywords

SARS-CoV-2; RdRp; Virtual screening; Pralatrexate; Azithromycin

Author Summary

Currently, a novel coronavirus called SARS-COV-2 is spreading across many parts of the world. Unfortunately, there is still a lack of effective drugs against the virus. Additionally, it usually takes much longer time to develop a new drug using traditional methods. Thus, it is now better to rely on some alternative methods to develop drugs that can treat such a disease effectively. In this paper, we have proposed a deep learning and molecular simulation dynamics based hybrid drug screening procedure for identifying potential drug candidates targeting RdRp from 1906 market available drugs. Our screening have successfully identified a FDA-approved drug called Pralatrexate that strongly inhibits the replication of 2019-nCoV in vitro with EC50 values of 0.008μM. This work demonstrated the feasibility of accurate virtual drug screening for inhibitors of SARS-CoV-2 and provides potential therapeutic agents against COVID-19.

Introduction

The Coronavirus Disease 2019 (COVID-19) caused by severe acute respiratory syndrome coronavirus 2 (SARS-CoV-2) has developed into a global pandemic with millions of people infected and tens of thousands of lives lost [1]. To date, no clinically proven drug or vaccine is available. There is an urgent need to identify antiviral agents that can inhibit SARS-CoV-2. De novo drug development process is time-consuming, unable to meet the urgent need to combat COVID-19. Given current emergencies, repurposing existing approved drugs for COVID-19 may provide a shortcut [2]. Drugs under recent clinical trials such as Remdesivir were shown to inhibit the replication of SARS-CoV-2 *in vitro* [3–5]. The structural basis of

the RNA-dependent RNA polymerase (RdRp) inhibited by Remdesivir is well illustrated in a recent work [6]. Some patients have been treated with compassionate-use of Remdesivir and shown significant clinical improvements [7]. As a core component of the RNA synthesis machinery, RdRp is believed to be one of the most promising therapeutic target [8,9]. Molecules that can bind to the catalytic site of RdRp could potentially interfere the viral RNA synthesis[10].

Several computational drug screening methods base on molecular docking, deep learning or Molecular Dynamics (MD) simulations have been applied in drug repositioning studies for COVID-19 [9,11–14]. However, most of these normally rely on a single technique or lacks experimental validation. Each computational method, with its underlying philosophy, often has its own strong and weak point, while a proper combination and modification of such methods could provide a better solution. Previously, we have developed two deep learning-based models to estimate protein-ligand interactions: DFCNN [15] and DeepBindBC (http://cbblab.siat.ac.cn/DeepBindBC/index.php). DFCNN uses molecular vector data of protein pocket and ligand, instead of spatial information at integration site, to estimates the protein-ligand pair as binding or non-binding with a probability value between 0 and 1. DeepBindBC estimates the binding possibility from atom contact information at interaction surface of a modelled 3D protein-ligand complex. The input of DeepBindBC contains spatial information of the protein-ligand interface, thus it strongly complements DFCNN.

We propose a hybrid screening procedure, based on deep learning and molecular simulation, consists of DFCNN [15], DeepBindBC, Autodock Vina [16], pocket localized molecular

dynamics simulation, and metadynamics, as well as our inhouse developed tools, to explore the binding potential of drugs from TargetMol-Approved_Drug_Library, a drug library containing 1906 of current market available drugs, by TargetMol, for possible repositioning of any drug under current global emergency situation. As a result, four drugs are considered possible interactor of RdRp and selected for further experimental validation, and Pralatrexate is identified as an effective inhibitor of replication of SARS-CoV-2 *in vitro*.

Results

A hybrid virtual screening procedure is performed, illustrated in Fig. 1**a**. 1906 approved drugs from TargetMol-Approved-Drug-Library were subject to the proposed screening process which consists of molecular vector-based screening, structure-based screening and force field-based screening. DFCNN and DeepBindBC are both deep learning-based methods. Four candidates (Pralatrexate, Azithromycin, Sofosbuvir, Amoxicillin) were selected by the proposed method for experimental validation. The qRT-PCR assay, indirect immunofluorescence assay (IFA) and CCK-8 assay were carried out to validate the efficacy for Pralatrexate, Azithromycin which inhibit SARS-CoV-2 replication *in vitro*. Surface plasmon resonance (SPR) assay was used to evaluate the RdRp-drug binding affinity.

Fig. 1 Drug repurposing against RdRp for COVID-19 using a hybrid deep learning and molecular simulation strategy. a, 1906 approved drugs were subject to the proposed screening process which consists of molecular vector-based screening, structure-based screening and force field-based screening. DFCNN and DeepBindBC are both deep learning-based methods. 4 candidate drugs were selected by the proposed method, including Pralatrexate, Azithromycin, Sofosbuvir, Amoxicillin. **b,** Key interactions between the studied

drugs and RdRp from the last frame of MD simulation, for (i) Azithromycin and (ii) Pralatrexate. RdRp binding pocket is shown in green with surface representation and the corresponding drugs are shown in magenta. The 2D Schematic diagram of drug-RdRp interaction is given bottom, and neighbor residues (within 4 Å of the drug) are shown. **b**(iii), the experimental structure of Remdesivir in its monophosphate form with RdRp (PDB ID 7BV2), the 2D Schematic diagram of the interaction was also shown.

Deep learning and docking

Interestingly, from Supplementary Table 2, we notice that DFCNN, DeepBindBC and Autodock Vina predict different drug compounds as top hits. This indicates that these three methods may potentially complementary to each other through judging protein-ligand interactions from different perspectives. For instance, Amenamevir and Azithromycin have better Autodock Vina scores whereas Odanacatib and Nitisinone are found to have high DFCNN scores.

We first rejected those drugs that had poor prediction by any of the methods. The 22 drugs with DFCNN score above 0.9 and docking score bellow -7 kcal/mol were firstly selected and presented in Supplementary Table 2. Among the 22 drugs, we further excluded the drugs with a DeepBindBC score below 0.7, resulting in 14 drugs shown in bold in Supplementary Table 2. Overall there are 5 nucleoside analogues, 2 antibiotic drugs, 3 antivirus drugs, 2 anticancer drugs and 3 other drugs (Supplementary Fig. 1) selected by the molecular vector-based and structure-based screening process. Sofosbuvir is both a nucleoside analogue and an antivirus drug. These 14 drugs are subject to force field-based screening in the next stage.

Among 2 antibiotic drugs, Azithromycin, a drug used to treat a variety of bacterial

infections, showed top Autodock Vina score of -8.6 kcal/mol, good DFCNN score and DeepBindBC score (0.9093 and 0.8589), respectively. Gautret, P. *et al* claimed that combined with Hydroxychloroquine, Azithromycin can have good efficiency in treating COVID-19 with significant viral load reduction [17]. However it should be noticed that currently there is no evidence of the effectiveness of Azithromycin in the treatment of COVID-19 and have many debates about effective of Azithromycin on COVID-19 [18].

The top two predicted molecules by DeepBindBC are nucleotide analogues. Sofosbuvir is a nucleotide analogue inhibitor of hepatitis C virus (HCV) NS5B polymerase to treat infectious liver disease, [19] whereas Clofarabine is a purine nucleoside antimetabolite used for treating refractory acute lymphoblastic leukaemia [20]. More nucleotides analogues in the candidate list were selected by our method, such as Adenosine, Vidarabine, and Gemcitabine, indicating some RdRp-nucleotides interaction patterns have been implicitly recognized by the proposed hybrid drug screening method.

Force field-based simulation

To further screen the 14 selected drugs (2D structures shown in Supplementary Fig. 1) and understand their interactions and stability, we have performed MD simulations on RdRp-drug complexes. The structure stability is estimated by Root Mean Square Deviation (RMSD) over 100ns time scales, shown in Supplementary Fig. 2. The drugs such as Azithromycin, Pralatrexate, Romidepsin, Teriflunomide and Vidarabine are found stable indicated by the minimum RMSD fluctuations. On the other hand, the drugs such as Adenosine, Amenamevir, Fipronil, Gemcitabine and Sofosbuvir have high RMSD fluctuations. The number of

hydrogen bonds formed between RdRp and the drug in the 100 ns MD simulation was also computed and shown in Supplementary Fig. 3. Among the 14 RdRp-drug complexes, Pralatrexate clearly showed more hydrogen bonds with the RdRp than others. We found that there is no direct correlation between RMSD fluctuation and size (or number of initial contacts). For instance, the Romidepsin has very large size (molecular weight: 544.7), but shows less binding stability compared to Pralatrexate (molecular weight: 479.5). Also, Vidarabine have molecular weight of 271.3, shows more stable binding compared to Sofosbuvir (molecular weight: 527) and Amenamevir (molecular weight: 484.6). In MD simulation, small size ligands can also have very tight binding depending on the ligand and binding site (pocket) properties [21]. Whether the binding is stable is more related to the complementary between pocket and ligand in both geometric and physio-chemical features. So the RMSD calculations can help to provide useful information about the binding stability.

The binding free energy *vs* coordination number (CV: collective variable) from metadynamics simulations is shown in Supplementary Fig. 4**a**. The lowest energy conformations of protein-drug complexes for Amoxicillin, Azithromycin, Pralatrexate and Sofosbuvir showed more contacts in the interface region, as indicated by the high coordination numbers (Supplementary Fig. 4**a** (iii)), while most other compounds favor smaller coordination number (close to zero) indicating no or weak interactions (Supplementary Fig. 4**a** (i)).

Detailed interaction patterns between RdRp and the four most optimal compounds (Azithromycin, Pralatrexate, Amoxicillin and Sofosbuvir) are shown in Fig. 1**b**(i)(ii) and Supplementary Fig. 4**b** (i)(ii), whose structures are taken from the last frame of the 100 ns

MD simulation. Azithromycin and Pralatrexate interact with 16 amino acid residues of RdRp to form a stable complex. The RdRp-Azithromycin complex is mainly dominated by van der Waals interactions, whereas Pralatrexate involves more polar and charge interactions. According to the calculated free energy difference (ΔG) values from the metadynamics simulations between the unbound state and the binding state for Amoxicillin, Azithromycin, Pralatrexate and Sofosbuvir (Supplementary Table 3), Azithromycin and Pralatrexate (-305.8 kJ/mol, -128.6 kJ/mol) show more favorable binding energy than Amoxicillin and Sofosbuvir (-67.3 kJ/mol and -89.9kJ/mol). Remdesivir has covalent bind with the RNA primer, our method can only estimate binding free energy between non-colvalent-binding protein and ligand. Furthermore, our system not contains RNA premier, so the interaction mechanism and pattern of the Pralatrexate and Azithromycin would be different from Remdesivir.

It is noted that all the nucleoside analogues highly recommended by deep learning-based screening methods were excluded from the force field based screening process. The possible explanation is that our protein-drug systems do not contain the RNA primers during the MD simulation, and without RNA the nucleoside analogues would not form base pair like interaction, hence shows no binding in MD simulation.

Interaction patterns (taken from the last frame of 100 ns MD simulation) between RdRp and Azithromycin, Pralatrexate are shown in Fig. 1**b**(i)(ii), respectively. The interaction between Remdesivir in its monophosphate form and RdRp (PDB ID: 7BV2) is also given for comparison in Fig. 1**b**(iii). Azithromycin forms 2 hydrogen bonds with GLN573 and ILE494 through keto and hydroxyl groups respectively, and many hydrophobic related interactions with the RdRp binding site (*e.g.* LYS577, LEU576, ALA685) through alkyl groups, whereas

Pralatrexate shows enhanced and more stable interactions with RdRp binding site, including 6 hydrogen bonds with GLN573, ARG569, ASN496, ASN497, LYS500 and GLY590. Pralatrexate also forms Alkyl or Pi-Alkyl interaction with LYS577 and LEU576, and salt bridges with ARG569 and LYS500. Azithromycin and Pralatrexate share 10 common neighbor residues (62.5%) of RdRp, as shown in Fig 1**b**(i) and **b**(ii) and Supplementary Table 1, indicating a similar binding cavity.

In a recent study, the authors have shown that the monophosphate active form of Remdesivir interacts with the RdRp and covalently incorporated into partial double stranded RNA template of RdRp at the +1 position [6]. Shown in Fig. 1**b**(iii) and Supplementary Table 1. The key interacting contacts between Remdesivir and RdRp include ARG555. Remdesivir only shares 2 common close contact residues with Pralatrexate, and no common close contact residue with Azithromycin, suggesting a different interaction pattern.

This screening procedure relies on non-covalent bond interactions and assumes RdRp is in apo form (without RNA primer), Figure 1**b**(i)(ii) shows Azithromycin and Pralatrexate interacting with the region of RdRp which is consist of residues such as GLN573, ARG569, ALA685. Comparing our modeled structure with Remdesivir in its monophosphate-RdRp complex, it is possible Azithromycin and Pralatrexate occupy part of the RdRp cavity with the non-covalent binding which may interfere the entry of the RNA primer strand to the cavity.

Pralatrexate and Azithromycin inhibit the replication of SARS-CoV-2 *in vitro*.

To further confirm the efficiency of the hits from the virtual screening, we tested the antiviral

activity of Azithromycin, Pralatrexate, Amoxicillin and Sofosbuvir *in vitro*. Experiments were performed in a biosafety level 3 laboratory where regulation requires to. Vero cells were infected with SARS-CoV-2 (BetaCoV/Shenzhen/SZTH-003/2020, GISAID No. EPI_ISL_406594) at a MOI of 0.02 (the cytopathic effect was mild at 48 hours post-infection with this MOI) in the presence of varying concentrations of the tested drugs, and the inhibition rates were evaluated by quantification of viral copy numbers in the cell supernatant via quantitative reverse transcription polymerase chain reaction reverse transcription polymerase chain reaction (qRT-PCR) and confirmed with immunofluorescence assay (Fig. 2). The results showed that Pralatrexate and Azithromycin could efficiently inhibit the replication of SARS-CoV-2, with half-maximal effective concentration (EC_{50}) values of 0.008 and 9.453 μM (Fig. 2**a**), whereas Remdesivir achieved an inhibitory activity with EC_{50} value of 8.777 μM within the same experimental system (Supplementary Fig. 5). IFA showed similar results with qRT-PCR assay (Fig. 2**b**). CCK-8 assay of the two drugs showed that the half-cytotoxic concentration (CC_{50}) values of Pralatrexate and Azithromycin on Vero cells were 0.167 μM and > 100 μM, respectively, and the calculated the selectivity indexes (SI) of Pralatrexate and Azithromycin were 20.878 and >10.579, respectively. Whether the two drugs worked at the stage of viral entry or post entry was analyzed using time-of-addition assay as previously reported[5]. The results showed that Pralatrexate functioned at a stage post virus entry, while Azithromycin functioned at both entry and post-entry stages of the SARS-COV-2 infection in Vero cells (Fig. 2**c**). Furthermore, surface plasmon resonance (SPR) experiments were performed to test the *in vitro* binding of Pralatrexate and Azithromycin with immobilized RdRp protein of SARS-CoV-2. Both drugs showed expected binding response

in Supplementary Fig. 6.

Fig. 2 The antiviral activities of the test drugs against SARS-COV-2 *in vitro*. a, Vero cells were infected with SARS-COV-2 at an MOI of 0.02 in the presence of the indicated concentrations of the tested drugs for 48 hours. The viral yield in the cell supernatant was then quantified by qRT-PCR. Meanwhile, cytotoxicity of these drugs to Vero cells was measured by CCK-8 assay. **b**, Immunofluorescence microscopy of virus infection upon treatment of Pralatrexate and Azithromycin at the indicated concentrations. IFA was performed at 48 hours post-infection. Scale bar, 100 μm. Cells were immunostained for the Viral protein (green) and DNA (blue). **c**, Time-of-addition experiment of Pralatrexate and Azithromycin. Cells were infected with SARS-COV-2 at an MOI of 0.02 with different treatment, and virus yield in the infected cell supernatants was quantified by qRT-PCR. For the group of “Entry”, the drugs were added to the cells for 1 hour before viral attachment, and at 2 hours post-infection, the virus-drug mixture was replaced with fresh culture medium. For the group of “Post-entry”, drugs were added at 2 hours post viral infection, and maintained until the end of the experiment. For the group of “Full-time”, Vero cells were pre-treated with the drugs for 1 hour, and the virus was then added to allow attachment for 2 hour. Afterwards, the virus-drug mixture was removed, and the cells were cultured with drug-containing medium until the end of the experiment.

Discussion

To perform the drug screening process efficiently and accurately is still a challenge for computer-aided drug design. Though a recent deep learning-based approaches has demonstrated its potential to be efficient/effective by learning from a sufficient amount of training data, problems such as overfitting, and the discrepancy between training data and real-world data remain [22]. The proposed deep learning and molecular simulation based

drug screening method was able to select 4 approved drug candidates targeting RdRp from 1906 drugs, and 2 out of 4 (Pralatrexate and Azithromycin) can effectively inhibit SARS-CoV-2 replication *in vitro* with EC_{50} values of 0.008μM and 9.453 μM. The molecular vector-based deep learning method and the structure-based deep learning method are complementary to each other in the sense that high efficiency and accuracy are both achieved. We noticed the experimental activities of Pralatrexate and Azithromycin do not correlate with the free energy of binding. The computational methods of free energy calculations include several limitations. This includes approximations involved for deriving force field parameters, and approximations in estimating energy contributions. An exact correlation between computational calculation and experimental free energy is not possible. Also the experimental activities may not always directly correlate with binding free energy, for instance, weak binding on critical residues may demonstrate better activity than binding on non-critical residues. Nonetheless, our computational calculations show good approximations when used in a screening context.

For the first time, Pralatrexate is found to potently inhibit SARS-CoV-2 replication *in vitro* with a stronger inhibitory activity (EC_{50} value： 0.008μM) than Remdesivir (P<0.0001) under same experimental setup. Compared with the GHDDI drug list (The Global Health Drug Discovery Institute: https://ghddi-ailab.github.io/Targeting2019-nCoV/preclinical/.) that inhibit SARS-CoV-2 *in vitro,* Pralatrexate showed the smallest EC_{50}. Among the 154 current reported drugs by GHDDI, NSC319726 have top inhibitory activity over SARS-CoV-2 (EC_{50} value <0.02μM)[23]. Pralatrexate is a folate analogue metabolic inhibitor, which was approved by FDA in 2009 for the treatment of patients with relapsed or refractory peripheral

T cell lymphoma (PTCL). Pralatrexate inhibits the folate metabolism pathway through inhibition of dihydrofolate reductase (DHFR) [24]. The peak concentration in plasma (Cmax) can achieve 10.5 μM from a standard dosing regimen [25]. Its Cmax is around 800-fold higher than the EC_{90} of antiviral activity, suggesting a great potential for clinical implications.

Pralatrexate was selected by the virtual screening pipeline based on its potential acts of inhibiting the RNA dependent RNA protease (RdRp) enzyme, whereas, its extremely low EC50 for the virus replication compared to Remdesivir (RdRp inhibitor) may have multiply mechanism of action involved as well. Pralatrexate is known to be an antifolate that efficiently prevents synthesis of DNA and presumably also RNA[26], which may explain inhibition of SARS-CoV-2 replication. Pralatrexate was approved by FDA for patient with terminal disease in spite of its toxicity, therefore, we should be aware that FDA approval does not guarantee the possibility of immediate use of the drug against COVID-19.

Though both Pralatrexate and Azithromycin inhibit SARS-CoV-2 replication *in vitro*, the time-of-addition experiment showed that they functioned at different stages of SARS-CoV-2 infection. Similar to Remdesivir, Pralatrexate mainly inhibited the replication of SARS-CoV-2 at the stages of post-entry. On the other hand, Azithromycin inhibited the replication of SARS-CoV-2 at both entry and post-entry stages like chloroquine [5]. This indicates the Azithromycin may also have multiple mechanism of action.

Out of the 4 selected drug candidates targeting RdRp, Amoxicillin and Sofasbuvir have failed to inhibit SARS-CoV-2 replication *in vitro.* Molecular dynamic simulations show they have deviated from its initial binding position (Supplementary Fig. 4 **a**(i) **a**(ii)) with their ligand RMSD > 1.5 nm for most of the simulation time and large fluctuation was observed

(Supplementary Fig. 2). The calculated free energies difference between binding state and unbound state (coordination number around 0) also indicates better binding for Pralatrexate and Azithromycin than Amoxicillin and Sofasbuvir, shown in Supplementary Fig. 4 a(iii) a(iv) and Supplementary Table 3.

To examine why Sofosbuvir can efficiently inhibit RdRp of hepatitis C virus (HCV)[19] while not RdRp of SARS-CoV-2, we have carried a sequence and structural comparison between RdRp of HCV and RdRp of SARS-CoV-2 virus (Supplementary Fig. 7). In addition to the low sequence identity (23.75%) between RdRp of HCV and RdRp of SARS-CoV-2 virus, binding pockets of both complexes showed a quite different composition. For instance, there are 5 *vs* 3 ASPs, 2 *vs* 1 LYSs, 1 *vs* 3 GLUs, 0 *vs* 6 ARGs in RdRp pocket of SARS-CoV-2 and RdRp pocket of HCV, respectively. The RdRp pocket of SARS-CoV-2 is more negatively charged, while the RdRp pocket of HCV is more positively charged.

Full system protein-ligand MD simulations for RdRp-Pralatrexate, RdRp-Azithromycin were performed to validate the robustness of the pocket MD method. Compared to pocket MD simulation, similar hydrogen bond numbers as well as similar low RMSD fluctuations in full MD simulation were observed according to Supplementary Fig. 3 and Supplementary Fig. 8**a**(i)(ii). Some key neighbor residues in pocket MD simulation for Azithromycin and Pralatrexate were also kept during the full system MD simulation, according to Fig. 1**b**(i)(ii) and Supplementary Fig. 8**b**(i)(ii). For instance, LEU576, ILE589, ALA580 and ALA685 have formed alkyl related hydrophobic interaction with Azithromycin in the last frames of both simulations, and ARG569, ASN496 and LYS500 of RdRp have formed salt bridge or hydrogen bonds with Pralatrexate in the last frames of both simulations.

The efficiency and effectiveness of the DFCNN method have been examined previously by screening about 10 million drugs targeting 8 representative protein targets taken from the DUD.E diverse data set. The running parameters of DFCNN are same as the current work. DFCNN was able to screen the 10 million drugs within 5 hours using only a workstation with 80 Intel CPU cores (2.00 GHz) and 60 GB RAM. The effectiveness is evaluated by the prediction-random ratio ($Ratio_{0.9}$), shown in Supplementary Table 4. For 6 out of 8 protein targets, $Ratio_{0.9}$ is greater than 1.4, indicating DFCNN is able to enrich the active compounds in ten million compound pools. Among the 8 test cases, the DFCNN achieved best performance on HIVPR (Human immunodeficiency virus type 1 protease) with $Ratio_{0.9}$ of ~860 (about 860 times better than random guess in selecting active compounds in terms of TPR). DFCNN performed worse for GPCR proteins (such as CXCR4) and protein with small inner pocket (such as AKT1). The possible reason is that GPCRs have limited number of reliable structure of protein-ligand complexes in our training dataset and membrane proteins may have very different binding mechanism compared to other type proteins. The poor performance for proteins with small inner pocket is likely due to the special physical-chemical and spatial features. As an enzyme, RdRp has large ligand binding cavity and should be suitable for virtual screening by DFCNN.

To study how the molecular vector-based deep learning screening method selects the 139 candidate drugs from 1906 drugs, 1906 drugs were clustered into 20 groups (Supplementary Fig. 9). Group 20 has the highest ratio of drugs being selected (31/89 drugs, Supplementary Fig. 9**a**). The drugs in the Groups 19, Groups 20, Group 17 and Group 15 with high selection ratio tend to contain many electron donors and electrical acceptors, likely due to the RdRp

pocket containing many charged groups (Supplementary Fig. 10), including 5 ASPs, 2 LYSs, and 1 GLU. The percentage of charge and polar residues in the RdRp pocket reaches 54.35% (Supplementary Fig. 10), which explains why DFCNN prefers to select polar and charged drugs for the RdRp. The structure-based screening (Autodock Vina plus DeepBindBC) selected 14 drugs from 139 drugs, 6 drugs belong to Group 15 (Supplementary Table 5), including nucleotide analogues as well as Pralatrexate. Groups 17, 18, 19 all have 2 drugs selected after structure-based screening. Belonging to different clusters, Pralatrexate has many hydrogen donors and acceptors while Azithromycin contains a macrocycle, which tends to form hydrogen bond (or salt bridge) and macrocyclic hydrophobic interactions, respectively.

Since Pralatrexate are known to inhibit DHFR, if it also inhibit RdRp as we assume in this paper, DHFR and RdRp binding pocket may show some similarities. To illustrate that, we extract the ligand binding pocket of DHFR (PDB ID 2W9G), and show it in Supplementary Table 12. Comparing with Supplementary Fig. 10b, we can clear see both pocket contains many charged residues (10 and 8, respectively), and polar residues (14 and 17, respectively), which all shows high percentage over the total residue number and indicates the hydrophilic of the pockets. And some type of residues have very similar number, for instance both pockets contains 5 ASP residues. The highly similar pocket physio-chemical feature may explain that the two pocket have share same inhibitor Pralatrexate.

Conclusion

Identifying effective drugs that can treat COVID-19 is important and urgent, especially the approved drugs that can be immediately tested in clinical trials. In this work, we have

developed a hybrid protocol of combining deep learning methods with molecular simulations to search for potential drug candidates against RdRp that can inhibit the replication of SARS-CoV-2. Four potential drugs were systematically selected for experimental validation, and Pralatrexate and Azithromycin showed an inhibiting effect with EC_{50} values of 0.008μM and 9.453 μM, respectively. Experimental results from qRT-PCR, CCK-8 assay, indirect immunofluorescence assay (IFA), Time-of-addition and Surface plasmon resonance (SPR) assay show the proposed screening protocol successfully identified a new therapeutic agent Pralatrexate against COVID-19 by targeting RdRp. The hybrid strategy of combining deep learning, molecular docking, MD simulation in a virtual screening pipeline can effectively help with drug repurposing application and facilitate virtual drug screening against other targets in SARS-CoV-2.

STAR★Methods

Structural modeling of RdRp and drug compound dataset

The RdRp sequence and its modelled structure were obtained from https://zhanglab.ccmb.med.umich.edu/C-I-TASSER/2019-nCov/. The RdRp-ligand model was constructed by I-TASSER[27]. The ligand was taken from the template protein (PDB ID: 3BR9)[28] by COFACTOR algorithm[29] within I-TASSER using structure comparison and protein-protein networks. We extract the amino acids within 1 nm of the ligand as the binding pocket. RMSD between the modeled structure and the recent experimental RdRp structure (PDB ID 6M71) is calculated (~0.516 Å), shown in Supplementary Fig. 11**a**)[10]. RNA polymerase superfamily region is also very similar between these two structures (RMSD=0.456 Å, Supplementary Fig. 11**a**).

TargetMol-Approved_Drug_Library, which contains 1906 compounds, was used as virtual screening library. These 1906 compounds collected by TargetMol are drugs approved by Food and Drug Administration (FDA), the European Medicine Agency (EMA), or China Food and Drug Administration (CFDA), or included in the US Pharmacopeia (USP) Dictionary, the British Pharmacopoeia (BP), the European Pharmacopoeia (EP), the Japanese Pharmacopoeia (JP), or Chinese Pharmacopoeia (CP) Dictionary.

Machine-learning based drug screening

DFCNN

A deep learning-based method, DFCNN (Dense fully Connected Neural Network), has been

developed for predicting protein-drug binding probability [15] and used in this paper for the initial drug screening (Fig. 1**a**). DFCNN utilizes the concatenated molecular vector of protein pocket and ligand as input representation, and the molecular vector are generated by Mol2vec[30] which is inspired by the word2vec model in natural language processing. DFCNN model was trained on a dataset extracted from PDBbind database [31]. Negative data samples in the dataset were generated by cross-combination of proteins and ligands from PDBbind database and positive data samples were taken from protein-ligand pairs in experimental structure. The details of the method were described in our previous paper [15], and DFCNN achieved an AUC value around 0.9 for the independent testing set [15]. The model is about ~100,000 times faster than Autodock Vina in predicting protein-ligand binding probability (range 0~1), because it does not rely on the protein-drug complex conformation.

We screen a large scale chemical compound dataset (about 10 million compounds) targeting 8 representative protein targets taken from the DUD.E diverse data set in order to examine the efficiency and effectiveness of the DFCNN method. For each target, the corresponding dataset contains some active compounds (between 40 and 536) in the DUD.E dataset and 10,402, 895 drug-like compounds from ZINC database. The effectiveness is measured by the prediction-random ratio ($Ratio_{0.9}$), defined as $TPR_{0.9}/Random_{0.9}$, where $TPR_{0.9}$ indicates the ratio ($N_{0.9}$/Active_num) between the number of active compounds with a DFCNN score larger than 0.9 ($N_{0.9}$) and the active number of compounds (Active_num). The total number of the compounds (Total_num) with score above 0.9 is defined as NN. The random selection rate ($Random_{0.9}$) is defined as NN/Total_num. Using cutoff score of 0.9, the prediction-random ratio measures the ratio of predicted TPR and random selection TPR.

DeepbindBC

DeepBindBC, an in-house deep learning-based software, is used for structure based drug screening. Unlike the DFCNN, the input of DeepBindBC includes both the physical-chemical information and spatial information between the protein-ligand interfaces (Fig. 1**a**), hence DeepBindBC is able to achieve higher accuracy, but requires protein-drug complex structure information as input generated by Autodock Vina.

Autodock Vina is used to dock the target with the potential ligands [16]. The pocket is determined by the location of ligand in the template protein (PDB ID: 3BR9) [28]. We set the cavity volume space with 3.5 nm, 3.5 nm and 3.5 nm in x, y, z dimensions from the pocket mass center. AutoDock Tools were used to convert PDB file format to PDBQT file format [32]. The exhaustiveness was set to 8; the num_modes was set to 20, and energy_range was set to 3. The scoring function and optimization algorithm of Autodock Vina have been well discussed in a previous article[16]. The TargetMol-Approved_Drug_Library is well prepared by the TargetMol Company in 3D with sdf format. We have prepared the docking ligand structure using standard procedure used in many drug discovery projects. Briefly, the steps are as follows. The ligand was converted from sdf to mol2 by Openbabel, and then converted into pdbqt by scripts in AutoDockTools. Since the Autodock Vina only considers the heavy atoms, and the polar hydrogens, the protonation state before docking is determined by the default methods in AutoDockTools. In this study, we selected the most likely targets for further validation by setting a binding energy threshold value of -7 kcal/mol.

The DeepBindBC is a ResNet model trained over the PDBbind database. In DeepBindBC, the protein-ligand interaction interface information will be converted into

figure-like metric, similar to DeepBindRG [22]. By incorporating the cross-docking (docking proteins and ligands from different experimental complexes) conformation as negative training data, DeepBindBC is highly possible to distinguish non-binders. Since DeepBindBC relies on docking conformation and DFCNN only uses molecular vector information, these two methods are complementary to each other and DeepBindBC takes much more time than DFCNN.

Pocket molecular dynamics and metadynamics

Further drug screening was carried out by force field based molecular dynamic (MD) simulations. The initial protein-drug complexes was from top score conformation Autodock Vina docking, the ligand was edited by pymol software [33] to make it in correct protonation state at pH 7. In this study, we selected 14 drug binding complexes for MD simulation, including Adenosine, Amenamevir, Amoxicillin, Azithromycin, Clofarabine, Fipronil, Gemcitabine, Nitisinone, Pralatrexate, Raltegravir, Romidepsin, Sofosbuvir, Teriflunomide and Vidarabine, respectively.

We also refined a pocket molecular dynamics simulation (pocket MD, Supplementary Fig. 11**b**) to facilitate the simulation process by only keeping the binding pocket region for simulation. Binding free energy calculation can be estimated by metadynamics simulations to explore whether protein-ligand will bind in solution. Metadynamics relies on addition of a bias potential to sample the free energy landscape along a specific collective variable of interest [34],[35]. Note that the binding free energy calculations from Metadynamics may only be suitable for detect the general trend of binding in virtual screening.

The pocket MD is same as the classical MD simulation, except that we only using the pocket region to reduce system size for simulation (Supplementary Fig. 11**b**), which is inspired by a previous dynamic undocking (DUck) method [36]. An in-house script was used to extract the pocket region of the protein (1nm within the binding ligand), the N terminal and C terminal ends were capped with the ACE and NHE terminals, respectively. We applied a position restrain to the ACE and NHE terminals to maintain the relative conformation of the pocket. MD simulation was carried out by Gromacs with AMBER-99SB force field [37,38]. The topology of ligand and the partial charges of ligand was generated by ACPYPE [39], which relies on Antechamber [40]. Firstly, we created a dodecahedron box and put the target-ligand complex at the center. A minimum distance from the protein to box edge was set to 1 nm. We filled the dodecahedron box with TIP3P water molecules [41], the counter ions were added to neutralize the total charge using the Gromacs program tool [42]. The long-range electrostatic interactions under the periodic boundary conditions was calculated with Particle Mesh Ewald approach [43]. A cutoff of 14 Å was used for van der Waals non-bonded interactions. Covalent bonds involving hydrogen atoms were constrained by applying the LINCS algorithm [44].

We performed the energy minimization steps with a step-size of 0.001ns, 100 ps simulation with isothermal-isovolumetric ensemble (NVT), and 10ns simulation with isothermal-isobaric ensemble (NPT) for water equilibrium. After that, a 100ns NPT production run (step size 2 fs) was carried out. The Parrinello-Rahman barostat and the modified Berendsen thermostat were used for simulation with a fixed temperature of 308 K and a pressure of 1 atm. RMSD and hydrogen bond number of the trajectory were calculated

using Gromacs tools.

The simulation was continued using the metadynamics approach for exploring the free energy landscape. The interface coordination number of atoms of protein ligand complex was used as collective variable (CV). The protein-ligand interface coordination numbers correlate with the numbers of atom contact, and larger coordination number usually indicates that protein-ligand is in binding state.

The coordination number C is defined as follows by Plumed:

$$C = \sum_{i \in A} \sum_{j \in B} S_{ij} \quad (1)$$ and

$$S_{ij} = \frac{1 - \left(\frac{r_{ij} - d_0}{r_0} \right)^n}{1 - \left(\frac{r_{ij} - d_0}{r_0} \right)^m} \quad (2)$$

In the simulation, n was 6, m was 12, d_0 was 0 nm and r_0 was 0.5 nm. d_0 is a parameter of the switching function. r_{ij} is the distance between atom i and atom j. The degrees of contacts between two groups of atoms can be estimated by above function(1) [45]. Metadynamics simulation for each protein-ligand system was performed for 100 ns (except protein-Azithromycin, which was extended to 300ns in order to reach the 0 Coordination Number and achieve convergences). During the metadynamics simulation, Gaussian values were deposited every 1 ps with a height of 0.3 kJ/mol. The widths of the Gaussians were 5 for the coordination number. The free energy landscapes of the metadynamics simulations along the CV were generated by the Plumed program and plotted using Gnuplot[46].

Tools used in analysis

The USCF Chimera, VMD, ICM-browser-Pro(http://www.molsoft.com/icm_browser_pro.html) and Discovery Studio Visualizer 2019 were used to generate the structure and to visualize the 2D protein-ligand interactions [47–49]. Clusfps (https://github.com/kaiwang0112006/clusfps) which depends on RDKit[50] was used to cluster the drugs in the dataset. The drug fingerprint was used as inputs with algorithm of Murtagh[51] being used for clustering 1906 drugs into 20 groups.

Cell culture

Vero cell (ATCC, CCL-81) was cultured at 37°C in Dulbecco's modified Eagle's medium (DMEM, Gibco) supplemented with 10% fetal bovine serum (FBS, Gibco) in the atmosphere with 5% CO_2. Cells were seeded in 96-well plates and cultured overnight with a density of 5 × 10^4 cells/well prior infection or drug feeding. Remdesivir, Azithromycin, Pralatrexat, Sofosbuvir and Amoxicillin were obtained from Selleck Chemicals. All drugs were dissolved in DMSO to prepare 50 mM stock solutions, and stored at -20℃. DMSO was used in the controls.

Viral stock titration by 50% tissue culture infective dose ($TCID_{50}$)

$TCID_{50}$ was measured as previously reported [52]. In brief, Vero cells in 96-well plates were grown to 80% confluence and infected with 10-fold serial dilutions of the stock SARS-CoV-2 (BetaCoV/Shenzhen/SZTH-003/2020, GISAID No. EPI_ISL_406594) for 1 h at 37°C. The inoculum was removed, and cells were overlaid with fresh DMEM plus 2% FBS. At 5 days post infection (d.p.i), plates were assessed for the lowest dilution in which 50% of the wells

exhibited cytopathic effects. The values of $TCID_{50}$ were calculated according to the Reed-Muench method[53].

Evaluation of antiviral activities of the drugs in Vero cells

Firstly, the cytotoxicity of the five drugs on Vero Cells were determined by CCK8 assays (Sangon). Then the antiviral activities of the drugs were evaluated as previously reported with some modification[5]. Vero cells seeded in 96-well plates were pre-treated with the different doses of the indicated drugs for 1 h, and then virus was subsequently added at multiplicity of infection (MOI) of 0.02 to allow infection for 2 h. Then, the virus-drug mixture was removed and cells were further cultured with fresh DMEM with 2% FBS and the indicated concentrations of drugs. At 48 hours post infection (h.p.i), the cell supernatant was collected and viral RNAs were extracted using the QIAamp RNA Viral Kit (Qiagen, Heiden, Germany) for further quantification analysis. The cells were collected for indirect immunofluorescence assay (IFA). All the experiments involving infectious SARS-CoV-2 were handled in BSL-3 facilities at the Shenzhen Third People's Hospital.

Quantitative reverse transcription polymerase chain reaction

This assay was carried out as described previously [54]. Viral RNAs were extracted from the samples using the QIAamp RNA Viral Kit (Qiagen, Heiden, Germany), and quantitative reverse transcription polymerase chain reaction (qRT-PCR) was performed using a commercial kit (Genrui-bio) targeting the S and N genes. The specimens were considered positive if the Ct value was $\leq$ 38.0, and negative if the results were undetermined. Specimens

with a Ct higher than 38 were repeated. The specimen was considered positive if the repeat results were the same as the initial result and between 38 and 40. If the repeat Ct was undetectable, the specimen was considered negative.

Indirect immunofluorescence assay (IFA)

IFA was carried out as previously reported [55,56]. Vero cells were fixed in 4% formaldehyde at 48 hours post infection. Then cells were permeabilized in 0.5% Triton X-100, blocked in 5% BSA in PBS, and then probed with the plasma of this patient or healthy control at a dilution of 1:500 for 1 h at room temperature. The cells were washed three times with PBS and then incubated with either goat anti-human IgG conjugated with Alexa fluor 488 at a dilution of 1:500 for 1 h (Invitrogen). The cells were then washed and stained with hoechest-33342 (Invitrogen) to detect nuclei. Fluorescence images were obtained and analyzed using EVOS FL Auto Imaging System (Invitrogen).

Protein expression and purification

The genes for nsp12 of SARS-CoV-2 isolate BetaCov/Wuhan/WH01/2019 (EPI_ISL_406798) was chemically synthesized with codon optimization for insect cells (*Spodoptera frugiperda*) by Synbio Technologies. The sequence was fused with a C-terminal thrombin cleavage site, a 6×His-tag and a 2×Strep-tag, and incorporated into pFastbac-1 plasmid. Recombinant protein was expressed with Hi5 cells at 27℃. Cells were harvested at 48 hpi（hour post infection）and resuspended in 25 mM HEPES pH 7.4, 1 M NaCl, 1 mM $MgCl_2$ and 2mM TCEP. An equal volume of the same buffer supplemented with 0.2% (v/v) Igepal CA-630 (Anatrace) was

added and incubated at 4 °C for 10 min. Cells were lysed by sonication and the lysate was clarified by ultracentrifugation. Cleared lysates were passed through a 0.22-μm filter film before further purification. The protein was purified by tandem affinity chromatography and SEC.

Surface plasmon resonance (SPR) assay

The affinities between nsp12 and drugs were measured at room temperature (r.t.) using a Biacore 8K system with CM5 chips (GE Healthcare). The nsp12 protein was immobilized on the chip with a concentration of 100 μg/mL (diluted by 0.1mM NaAc, PH 4.0).

Drug samples were prepared according to procedure 29264621AA of GE Healthcare Life Sciences. 1×PBS solution plus 5% DMSO and 0.005% p20 was used for running and diluting drugs. A blank channel of the chip was used as the negative control. Serial diluted drugs were then flowed through the chip surface. The LMW multi-cycle kinetics was analyzed with the Biacore 8K Evaluation Software (version1.1.1.7442) and fitted with a 1:1 binding model.

Statistical analysis

Data are presented as the mean ± SD (Standard Deviation). All analyses were performed using GraphPad Prism version 7.0 for Windows (GraphPad Software, San Diego California, USA). Data were subjected to statistical analysis by two-way ANOVA or two-tailed Student's t-test. The P values less than 0.05 were considered statistically significant.

DATA AVAILABILITY

The structures for SARS-CoV-2 RdRp have been retrieved from the Protein Data Bank with accession ID 6M71, 6WTT. The template structure we used to construct RdRp-ligand complexe is retrieved from the Protein Data Bank with accession ID 3BR9). We have deposited all data underlying the figures and results into the github (https://github.com/haiping1010/SARS_RdRp_drug_screening_data) for the convenience of readers. The MD trajectory and some original experimental data which are too large to deposited in github was putted in figshare.com with a download link (https://figshare.com/s/c63312c68952cdf6b586).

Acknowledgement

Thanks to TargetMol for providing the approved drug library. This work was partly supported by Strategic Priority CAS Project XDB38000000, the National Key Research and Development Program of China under Grant No. 2018YFB0204403, 2016YFB0201305 and 2019YFA0906100, National Science and Technology Major Project under Grant No. 2018ZX10101004, Shenzhen Basic Research Plan under Grant No. JCYJ20190807170801656, National Science Foundation of China under Grant no. U1813203 and 61433012; the National Natural Youth Science Foundation of China (Grant no. 31601028), the Shenzhen Basic Research Fund under Grant no. JCYJ20180507182818013, JCYJ20170413093358429 and GGFW2017073114031767, the China Postdoctoral Science Foundation under Grant no. 2019M653132, CAS Key Lab under Grant no. 2011DP173015, and the SIAT Innovation Program for Excellent Young Researchers. We would also like to thank the funding support by the Shenzhen Discipline Construction Project for Urban

Computing and Data Intelligence, Youth Innovation Promotion Association, CAS to Yanjie Wei. Lots thanks to Harvey Wang from Xiamen Novel AIdrug Co.,LTD for his valuable advices and helps.

Supplementary information

S1 File. Supplementary Material.

Author contributions

H.Z. conceived the original idea, designed and implemented the hybrid virtual screening method, performed the drug screening step, analyzed the results, and participated in manuscript preparation. Y.Y. and J.W. tested inhibitory activity with qRT-PCR, CCK-8 assay, indirect immunofluorescence assay (IFA), Time-of-addition experiment and contributed to the analysis of the results and to the writing of the manuscript. J.L. participated in manuscript preparation and supervised the experimental process. M.W. helped with the manuscript writing and tested the drug-RdRp binding with Surface plasmon resonance (SPR). S.K., N.J., M.H., M.L., H.Z., Y.P., X.R. and Y.P. helped with manuscript preparation. Y.W., Y.S., X.W., and Y.L. supervised the project, and corrected the manuscript.

Competing interests

The authors declare no competing interests.

REFERENCES

1. Wu F, Zhao S, Yu B, Chen YM, Wang W, Song ZG, et al. A new coronavirus associated with human respiratory disease in China. Nature. 2020. doi:10.1038/s41586-020-2008-3

2. Zhou Y, Hou Y, Shen J, Huang Y, Martin W, Cheng F. Network-based drug repurposing for novel coronavirus 2019-nCoV/SARS-CoV-2. Cell Discov. 2020. doi:10.1038/s41421-020-0153-3

3. Caly L, Druce JD, Catton MG, Jans DA, Wagstaff KM. The FDA-approved Drug Ivermectin inhibits the replication of SARS-CoV-2 in vitro. Antiviral Res. 2020; 104787. doi:https://doi.org/10.1016/j.antiviral.2020.104787

4. Choy K-T, Wong AY-L, Kaewpreedee P, Sia SF, Chen D, Hui KPY, et al. Remdesivir, lopinavir, emetine, and homoharringtonine inhibit SARS-CoV-2 replication in vitro. Antiviral Res. 2020;178: 104786. doi:https://doi.org/10.1016/j.antiviral.2020.104786

5. Wang M, Cao R, Zhang L, Yang X, Liu J, Xu M, et al. Remdesivir and chloroquine effectively inhibit the recently emerged novel coronavirus (2019-nCoV) in vitro. Cell Research. 2020. doi:10.1038/s41422-020-0282-0

6. Yin W, Mao C, Luan X, Shen D-D, Shen Q, Su H, et al. Structural basis for inhibition of the RNA-dependent RNA polymerase from SARS-CoV-2 by remdesivir. Science (80-). 2020. doi:10.1126/science.abc1560

7. Grein J, Ohmagari N, Shin D, Diaz G, Asperges E, Castagna A, et al. Compassionate Use of Remdesivir for Patients with Severe Covid-19. N Engl J Med. 2020. doi:10.1056/NEJMoa2007016

8. Kirchdoerfer RN, Ward AB. Structure of the SARS-CoV nsp12 polymerase bound to

nsp7 and nsp8 co-factors. Nat Commun. 2019. doi:10.1038/s41467-019-10280-3

9. Wu C, Liu Y, Yang Y, Zhang P, Zhong W, Wang Y, et al. Analysis of therapeutic targets for SARS-CoV-2 and discovery of potential drugs by computational methods. Acta Pharm Sin B. 2020. doi:10.1016/j.apsb.2020.02.008

10. Gao Y, Yan L, Huang Y, Liu F, Zhao Y, Cao L, et al. Structure of the RNA-dependent RNA polymerase from COVID-19 virus. Science (80-). 2020; eabb7498. doi:10.1126/science.abb7498

11. Kumar Y, Singh H, Patel CN. In silico prediction of potential inhibitors for the main protease of SARS-CoV-2 using molecular docking and dynamics simulation based drug-repurposing. J Infect Public Health. 2020. doi:10.1016/j.jiph.2020.06.016

12. Maffucci I, Contini A. In Silico Drug Repurposing for SARS-CoV-2 Main Proteinase and Spike Proteins. J Proteome Res. 2020. doi:10.1021/acs.jproteome.0c00383

13. Trezza A, Iovinelli D, Santucci A, Prischi F, Spiga O. An integrated drug repurposing strategy for the rapid identification of potential SARS-CoV-2 viral inhibitors. Sci Rep. 2020. doi:10.1038/s41598-020-70863-9

14. Cavasotto CN, Di Filippo JI. In silico Drug Repurposing for COVID-19: Targeting SARS-CoV-2 Proteins through Docking and Consensus Ranking. Mol Inform. 2020. doi:10.1002/minf.202000115

15. Zhang H, Liao L, Cai Y, Hu Y, Wang H. IVS2vec: A tool of Inverse Virtual Screening based on word2vec and deep learning techniques. Methods. 2019. doi:10.1016/j.ymeth.2019.03.012

16. Brooks BR, III CLB, A. D. Mackerell J, Nilsson L, Petrella RJ, Roux B, et al.

Autodock Vina. J Comput Chem. 2009. doi:10.1002/jcc

17. Gautret P, Lagier J-C, Parola P, Hoang VT, Meddeb L, Mailhe M, et al. Hydroxychloroquine and azithromycin as a treatment of COVID-19: results of an open-label non-randomized clinical trial. Int J Antimicrob Agents. 2020. doi:10.1016/j.ijantimicag.2020.105949

18. Gbinigie K, Frie K. Should azithromycin be used to treat COVID-19? A rapid review. BJGP Open. 2020. doi:10.3399/bjgpopen20x101094

19. Bhatia HK, Singh H, Grewal N, Natt NK. Sofosbuvir: A novel treatment option for chronic hepatitis C infection. J Pharmacol Pharmacother. 2014. doi:10.4103/0976-500X.142464

20. Faderl S, Ferrajoli A, Wierda W, Huang X, Verstovsek S, Ravandi F, et al. Clofarabine combinations as acute myeloid leukemia salvage therapy. Cancer. 2008. doi:10.1002/cncr.23816

21. Barros EP, Schiffer JM, Vorobieva A, Dou J, Baker D, Amaro RE. Improving the Efficiency of Ligand-Binding Protein Design with Molecular Dynamics Simulations. J Chem Theory Comput. 2019. doi:10.1021/acs.jctc.9b00483

22. Zhang H, Liao L, Saravanan KM, Yin P, Wei Y. DeepBindRG: A deep learning based method for estimating effective protein-ligand affinity. PeerJ. 2019. doi:10.7717/peerj.7362

23. Bernhard Ellinger, Denisa Bojkova, Andrea Zaliani, Jindrich Cinatl, Carsten Claussen, Sandra Westhaus, Jeanette Reinshagen, Maria Kuzikov, Markus Wolf, Gerd Geisslinger, Philip Gribbon SC. Identification of inhibitors of SARS-CoV-2 in-vitro

cellular toxicity in human (Caco-2) cells using a large scale drug repurposing collection CURRENT STATUS : UNDER REVIEW. : 1–19. doi:10.21203/rs.3.rs-23951/v1

24. Ito Y, Makita S, Tobinai K. Development of new agents for peripheral T-cell lymphoma. Expert Opinion on Biological Therapy. 2019. doi:10.1080/14712598.2019.1572746

25. Foss FM. Evaluation of the pharmacokinetics, preclinical and clinical efficacy of pralatrexate for the treatment of T-cell lymphoma. Expert Opinion on Drug Metabolism and Toxicology. 2011. doi:10.1517/17425255.2011.595404

26. Kompis IM, Islam K, Then RL. DNA and RNA synthesis: Antifolates. Chemical Reviews. 2005. doi:10.1021/cr0301144

27. Zhang Y. I-TASSER server for protein 3D structure prediction. BMC Bioinformatics. 2008. doi:10.1186/1471-2105-9-40

28. Zhou Y, Webber SE, Murphy DE, Li LS, Dragovich PS, Tran C V., et al. Novel HCV NS5B polymerase inhibitors derived from 4-(1′,1′-dioxo-1′,4′-dihydro-1′λ6-benzo[1′,2′,4′]thiadiazin-3′-yl)-5-hydroxy-2H-pyridazin-3-ones. Part 1: Exploration of 7′-substitution of benzothiadiazine. Bioorganic Med Chem Lett. 2008;18: 1413–1418. doi:https://doi.org/10.1016/j.bmcl.2008.01.007

29. Roy A, Yang J, Zhang Y. COFACTOR: An accurate comparative algorithm for structure-based protein function annotation. Nucleic Acids Res. 2012. doi:10.1093/nar/gks372

30. Jaeger S, Fulle S, Turk S. Mol2vec: Unsupervised Machine Learning Approach with

Chemical Intuition. J Chem Inf Model. 2018. doi:10.1021/acs.jcim.7b00616

31. Liu Z, Li Y, Han L, Li J, Liu J, Zhao Z, et al. PDB-wide collection of binding data: Current status of the PDBbind database. Bioinformatics. 2015. doi:10.1093/bioinformatics/btu626

32. Morris GM, Ruth H, Lindstrom W, Sanner MF, Belew RK, Goodsell DS, et al. Software news and updates AutoDock4 and AutoDockTools4: Automated docking with selective receptor flexibility. J Comput Chem. 2009. doi:10.1002/jcc.21256

33. DeLano WL. Pymol: An open-source molecular graphics tool. CCP4 Newsl Protein Crystallogr. 2002.

34. Laio A, Gervasio FL. Metadynamics: A method to simulate rare events and reconstruct the free energy in biophysics, chemistry and material science. Reports Prog Phys. 2008. doi:10.1088/0034-4885/71/12/126601

35. Saleh N, Ibrahim P, Saladino G, Gervasio FL, Clark T. An Efficient Metadynamics-Based Protocol to Model the Binding Affinity and the Transition State Ensemble of G-Protein-Coupled Receptor Ligands. J Chem Inf Model. 2017. doi:10.1021/acs.jcim.6b00772

36. Ruiz-Carmona S, Schmidtke P, Luque FJ, Baker L, Matassova N, Davis B, et al. Dynamic undocking and the quasi-bound state as tools for drug discovery. Nat Chem. 2017. doi:10.1038/nchem.2660

37. Hess B, Kutzner C, Spoel D Van Der. GROMACS 4: algorithms for highly efficient, load-balanced, and scalable molecular simulation. J Chem. 2008.

38. Hornak V, Simmerling C. Generation of accurate protein loop conformations through

low-barrier molecular dynamics. Proteins Struct Funct Genet. 2003. doi:10.1002/prot.10363

39. Sousa Da Silva AW, Vranken WF. ACPYPE - AnteChamber PYthon Parser interfacE. BMC Res Notes. 2012. doi:10.1186/1756-0500-5-367

40. Wang J, Wang W, Kollman PA, Case DA. Automatic atom type and bond type perception in molecular mechanical calculations. J Mol Graph Model. 2006. doi:10.1016/j.jmgm.2005.12.005

41. Jorgensen WL, Chandrasekhar J, Madura JD, Impey RW, Klein ML. Comparison of simple potential functions for simulating liquid water. J Chem Phys. 1983. doi:10.1063/1.445869

42. Van Der Spoel D, Lindahl E, Hess B, Groenhof G, Mark AE, Berendsen HJC. GROMACS: Fast, flexible, and free. Journal of Computational Chemistry. 2005. doi:10.1002/jcc.20291

43. Darden T, York D, Pedersen L. Particle mesh Ewald: An N·log(N) method for Ewald sums in large systems. J Chem Phys. 1993. doi:10.1063/1.464397

44. Hess B, Bekker H, Berendsen HJC, Fraaije JGEM. LINCS: A Linear Constraint Solver for molecular simulations. J Comput Chem. 1997. doi:10.1002/(SICI)1096-987X(199709)18:12<1463::AID-JCC4>3.0.CO;2-H

45. Tribello GA, Bonomi M, Branduardi D, Camilloni C, Bussi G. PLUMED 2: New feathers for an old bird. Comput Phys Commun. 2014. doi:10.1016/j.cpc.2013.09.018

46. Williams T, Kelley C, Campbell J, Cunningham R, Denholm D, Elber G, et al. Gnuplot 4.6. Softw Man. 2012.

47. Pettersen EF, Goddard TD, Huang CC, Couch GS, Greenblatt DM, Meng EC, et al. UCSF Chimera - A visualization system for exploratory research and analysis. J Comput Chem. 2004. doi:10.1002/jcc.20084

48. Humphrey W, Dalke A, Schulten K. VMD: Visual molecular dynamics. J Mol Graph. 1996. doi:10.1016/0263-7855(96)00018-5

49. Visualizer DS. v4. 0.100. 13345. Accelrys Software Inc. 2005.

50. Landrum G. RDKit: Open-source Cheminformatics. In: Http://Www.Rdkit.Org/. 2006. doi:10.2307/3592822

51. Murtagh F, Contreras P. Algorithms for hierarchical clustering: An overview. Wiley Interdiscip Rev Data Min Knowl Discov. 2012. doi:10.1002/widm.53

52. Yang Y, Wong G, Ye B, Li S, Li S, Zheng H, et al. Development of a reverse transcription quantitative polymerase chain reaction-based assay for broad coverage detection of African and Asian Zika virus lineages. Virol Sin. 2017. doi:10.1007/s12250-017-3958-y

53. Reed LJ, Muench H. A simple method of estimating fifty per cent endpoints. Am J Epidemiol. 1938. doi:10.1093/oxfordjournals.aje.a118408

54. Zhao F, Yang Y, Wang Z, Li L, Liu L, Liu Y. The Time Sequences of Oral and Fecal Viral Shedding of Coronavirus Disease 2019 (COVID-19) Patients. Gastroenterology. 2020. doi:10.1053/j.gastro.2020.05.035

55. Yang Y, Zhang L, Geng H, Deng Y, Huang B, Guo Y, et al. The structural and accessory proteins M, ORF 4a, ORF 4b, and ORF 5 of Middle East respiratory syndrome coronavirus (MERS-CoV) are potent interferon antagonists. Protein Cell.

2013. doi:10.1007/s13238-013-3096-8

56. Yang Y, Ye F, Zhu N, Wang W, Deng Y, Zhao Z, et al. Middle East respiratory syndrome coronavirus ORF4b protein inhibits type i interferon production through both cytoplasmic and nuclear targets. Sci Rep. 2015. doi:10.1038/srep17554

Figure 1

Click here to access/download;Figure;Figure_1.tif

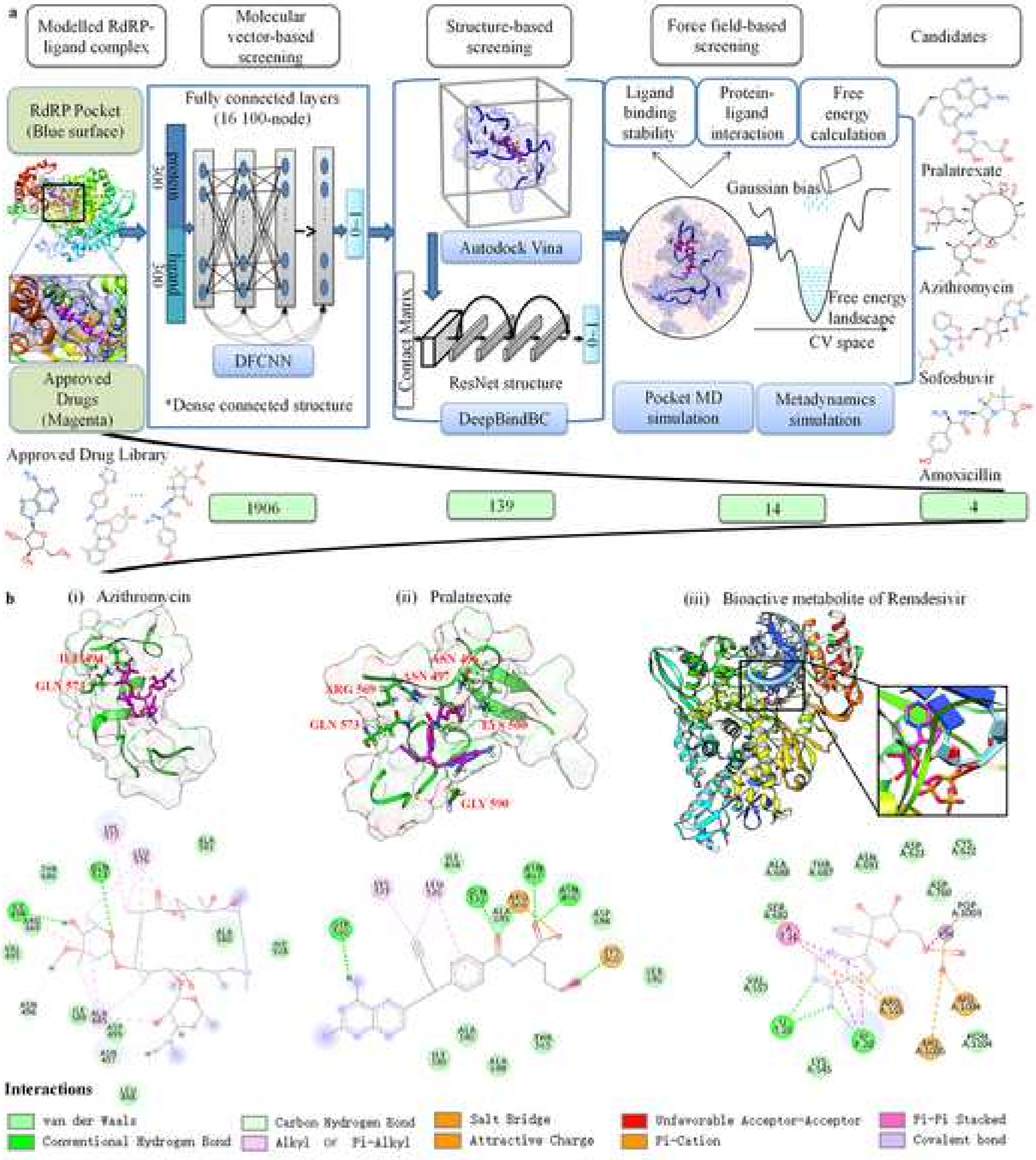

Figure 2

[Click here to access/download;Figure;Figure_2.tif]

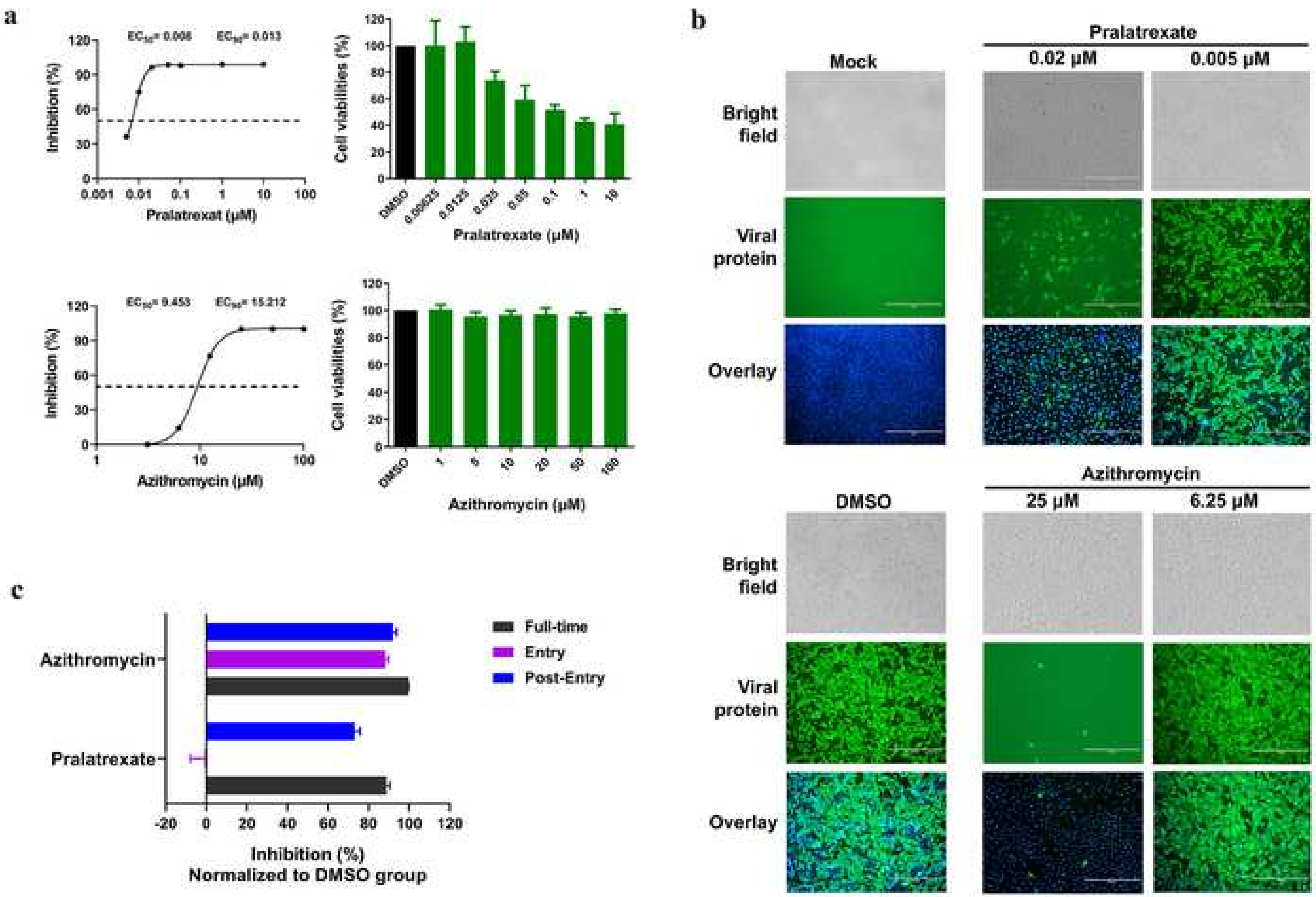

S1 file

Click here to access/download
Supporting Information
Supplementary Material.docx

Related Manuscript (marked-up copy)

Click here to access/download
Related Manuscript
manuscript-with-mark.docx
