# Supplementary material for: A novel virtual screening procedure identifies Pralatrexate as inhibitor of SARS-CoV-2 RdRp and it reduces viral replication in vitro
Source: PLoS Comput Biol. 2020 Dec 31;16(12):e1008489. doi: 10.1371/journal.pcbi.1008489 (PMC7774833; doi:10.1371/journal.pcbi.1008489)
Supplement: Attachment — Submitted filename: corresponds_to_reviewers.docx [file pcbi.1008489.s018.docx]

**Reviewer: 1**

**Comments**

The authors report an interesting study involving various *in silico* approaches for drug repositioning and experimental validation of some selected compounds. In my opinion some sentences need some additional explanations. My points are reported below:

Preparation of TargetMol-Approved_Drug_Library in 3D for docking?

Protonation state? 3D structure generator..? no warning / flag alerts for highly toxic compounds ?

When docking with Autodock Vina, the authors keep the best pose or they also investigate some top poses for each ligand as, the best energy pose does not mean that this is true pose in the binding pocket

I do not understand this sentence in the method: clustering 1906 drugs into 20 groups? There should be much more than 20 groups? or it depends how the groups are defined.

Could it be that Azithromycin has some good docking score only because it is big?

Could it be that drugs that are small or making less contacts have high RMSD fluctuations ?and if so, the type of simulation used mainly confirm that a large compound with many polar interactions will stick to a protein while a small compound will be unstable… If so, the tool does not discriminate anything? Please clarify or comment

I do not understand these sentences:

starting line: 177:

The possible explanation is that our protein-drug systems do not contain the RNA primers during the MD simulation, and covalent bond formation, such as the Remdesivir in its monophosphate form, could not be estimated by traditional MD simulation.

Few lines after, if some of the drugs used here are covalent binders, this should be explained further, please optimize the sentence

The authors suggest that Pralatrexate is already known to be active on Cov2 (as seen in the GHDDI list) but that in that list, the molecular mechanism of action of Pralatrexate has never been described or never shown to involve the RdRp target? Is it correct? and thus that Pralatrexate not only inhibits DHFR but also RdRp? If so, is there some similarity between the binding site of both targets? Please comment

>>Preparation of TargetMol-Approved_Drug_Library in 3D for docking? Protonation state ? 3D structure generator..? no warning / flag alerts for highly toxic compounds ?

Response: These are very good points. The TargetMol-Approved_Drug_Library is well prepared by the TargetMol Company in 3D with sdf format. We have prepared the docking ligand structure using standard procedure used in many drug discovery projects. Briefly, the steps are as follows. The ligand was converted from sdf to mol2 by Openbabel, and then converted into pdbqt by scripts in AutoDockTools. Since the Autodock Vina only considers the heavy atoms, and the polar hydrogens, the protonation state before docking is determined by the default methods in AutoDockTools. It should be noted that before MD simulation, we carefully visualized and checked each docked ligand manually to make sure they are in correct protonation state by pymol software [1]. At this first stage, we haven’t considered whether a compound is highly toxic or not because the most of compounds in this dataset are FDA approved drugs, having passed human toxicity tests prior to approval. We hence assumed they should be clinically safe for human uses. We have added this detailed information in Line 471-477 and Line 491-493 of the revised manuscript.

>>When docking with Autodock Vina, the authors keep the best pose or they also investigate some top poses for each ligand as, the best energy pose does not mean that this is true pose in the binding pocket

Response: Thanks for your helpful advice. Exactly, we considered this when using DeepBindBC, so we estimated and analyzed each of the generated docking poses, and then choose the top score as the final score. Here, we used the best Autodock Vina score corresponding pose as initial conformation for the next stage MD simulation. The best docking score pose is not guaranteed to be always correct as you suggested. However, the candidates of interest were subjected to MD simulation later, and MD simulation itself may be able to sample the correct binding pose if the initial structure dose not deviated too large. Usually, the last frame of MD simulation or the representative conformation of the largest cluster is more likely to be the correct binding pose than Docking.

>>I do not understand this sentence in the method: clustering 1906 drugs into 20 groups ? There should be much more than 20 groups? or it depends how the groups are defined.

Response: Thanks for your helpful advice. Many clustering methods require an initial arbitrary definition of number of clusters or cutoff values. In this study, we used the algorithm of Murtagh[2] by Clusfps (https://github.com/kaiwang0112006/clusfps) to do the clustering. This method requires the users to define the group number. We have tried different value of group number, and find 20 group can generate groups member with very similar drug properties (charge, polar, size, or hydrophobic, *etc*). We also attempt to do clustering with the algorithm of Butina [3], in which no need extra cluster number definition. But it still requires to define a cutoff value, which is also some arbitrary.

>>Could it be that Azithromycin has some good docking score only because it is big?

Response: Very good point, thanks for the concerns. The Docking score function sometimes favors hydrophobic interaction, and selects compound with large size according to our previous study [4] and other literature reports[5]. That is why we usually won’t use docking score as the only criterion to select drug candidates. It can be noticed that Azithromycin also has very good DFCNN and DeepBindBC scores which have no bias on hydrophobic interaction. Furthermore, Azithromycin is also stable in MD simulation，and favors binding on the pocket in metadynamics based free energy calculation. Because several methods we used all predict Azithromycin likely to binding with RdRp pocket, we choose it for final experimental validation.

>>Could it be that drugs that are small or making less contacts have high RMSD fluctuations? and if so, the type of simulation used mainly confirm that a large compound with many polar interactions will stick to a protein while a small compound will be unstable… If so, the tool does not discriminate anything ? Please clarify or comment

Response: Thanks for your helpful advice. We have checked the RMSD fluctuations of drugs that are small or with fewer contacts as you suggested. And we found that there is no direct correlation between RMSD fluctuation and size (or number of initial contacts). For instance, the Romidepsin has very large size (molecular weight: 544.7), but shows less binding stability compared to Pralatrexate (molecular weight: 479.5). Also, Vidarabine have molecular weight of 271.3, shows more stable binding compared to Sofosbuvir (molecular weight: 527) and Amenamevir (molecular weight: 484.6). In MD simulation, small size ligands can also have very tight binding depending on the ligand and binding site (pocket) property[6]. Whether the binding is stable is more related to the complementary between pocket and ligand in both geometric and physio-chemical features. So the RMSD calculations can help to provide useful information about the binding stability. It should be noted that we use the calculated free energy to do the final decision about which compounds should be chosen for last stage experimental validation, while the ligand RMSD in the MD simulation are not so reliable for judging whether a drug is a good candidates in some cases, especially when the initial binding conformation is deviated from the native one. We have clarified it in the Line 174-183 with above sentences.

>>I do not understand these sentences:

starting line: 177:

The possible explanation is that our protein-drug systems do not contain the RNA primers during the MD simulation, and covalent bond formation, such as the Remdesivir in its monophosphate form, could not be estimated by traditional MD simulation.

Few lines after, if some of the drugs used here are covalent binders, this should be explained further, please optimize the sentence

Response: Thanks for your helpful advice. We have not used any drugs that are covalent binders. Yes, it is quite easy to mislead by using this sentence. We have changed this sentence into “The possible explanation is that our protein-drug systems do not contain the RNA primers during the MD simulation, and without RNA the nucleoside analogues would not form base pair like interaction, hence shows no binding in MD simulation.” in Line 208-210 of the revised manuscript.

>>The authors suggest that Pralatrexate is already known to be active on Cov2 (as seen in the GHDDI list) but that in that list, the molecular mechanism of action of Pralatrexate has never been described or never shown to involve the RdRp target ? Is it correct ?and thus that Pralatrexate not only inhibits DHFR but also RdRp ? If so, is there some similarity between the binding site of both targets ? Please comment

Response: Thanks for your helpful advice. No, only the Azithromycin is already known to be active on inhibiting SARS-CoV-2 replication (as seen in the GHDDI list). But in that list, the molecular mechanism of action of Azithromycin has never been described or never shown to involve the RdRp target. For the Pralatrexte, our study is the first time to find its potent inhibition on SARS-CoV-2 replication. Pralatrexate is known to inhibit DHFR before and our study indicates it also inhibits RdRp to reduce the SARS-CoV-2 replication.

Thanks for the helpful advice about checking the binding site similarity between DHFR and RdRp. Interestingly, we found some similarities after careful investigation, for instance both the ligand binding pockets of DHFR and RdRp contain many charged and polar residues, and have similar number of different charged residues, *e.g.* both contains 5 ASP residues. And we added a paragraph to describe this in Line 390-398, and added a Supplementary Figure 12.

**Reviewer: 2**

**Comments**

The manuscript by Zhang et al. presents very interesting results obtained by applying different in silico methods, followed by reliable experimental validation. Methods are fine and results are well presented. However, some stylistic improvements are needed:

1. In the title: the viral titer is defined as the lowest concentration of virus (number of infectious units / ml) that can still infects cells. Lowering the viral titer actually means "making the virus more virulent", since a lower virus concentration is needed to infect the cell. I suggest changing in "Reduces Viral Replication" or something similar.

2. Lines 69-70: the authors states that "Several computational drug screening methods..." but only cite their own work. Please include some other citation (from a quick googling: https://doi.org/10.1016/j.jiph.2020.06.016; https://doi.org/10.1021/acs.jproteome.0c00383; https://www.nature.com/articles/s41598-020-70863-9; https://doi.org/10.1002/minf.202000115 )

3. Fig. 1: please edit figures to make text readable (I'm no more in my 20th, but reading was hard even when wearing glasses...)

4. Lines 118-121: the paragraph (from "Molecular" to "top hits") needs to be rephrased for better clarity.

5. Line 123: "rejected"..."that had poor..."

6. Lines 168-169: van der Waals

7. Line 170: "metadynamics"

8. Lines 173-174: energies in kJ/mol should be rounded to one decimal place

9: Lines 192-194: the paragraph (from "A recent" to "Table 1") needs to be rephrased for better clarity.

10. Line 200: "interacting....which is consist of"

11. Line 202: "it is possible that Azithromycin and Pralatrexate occupy"

12. Line 208: "activity of Azithromycin,..."

13. Line 229: I would not say that the results is "obvious" (maybe "expected")

14. Lines 275-276 "its extremely low EC50"

15. Line 334: "many electron donors and acceptors"

16. Line 367: "within I-TASSER"

17. Lines 394-404: do the reported parameters belong to the current work or are them from ref 11? Please only report relevant information

18. Line 414: "PDB"

19. Line 439: "Note that the..."

20. Lines 445-446: from "Terminals will" to "pocket" needs rephrasing21: Line 451: "ions were added"

22. Lines 459-460: check spacing

23. Lines 467-468: "indicates that protein-lingand..."

24. Line 474: which "function"?

25. Line 574: "Thanks to TargetMol for providing"

26. Line 829. "The physico-chemical"

>>1. In the title: the viral titer is defined as the lowest concentration of virus (number of infectious units / ml) that can still infects cells. Lowering the viral titer actually means "making the virus more virulent", since a lower virus concentration is needed to infect the cell. I suggest changing in "Reduces Viral Replication" or something similar.

Response: Thanks for the good comments. Yes, previous title will lead to misunderstanding. We have revised the title into “A Novel Virtual Screening Procedure Identifies Pralatrexate as Inhibitor of SARS-CoV-2 RdRp and It Reduces Viral Replication in vitro” in the revised manuscript according to your helpful suggestion.

>>2. Lines 69-70: the authors states that "Several computational drug screening methods..." but only cite their own work. Please include some other citation (from a quick googling: https://doi.org/10.1016/j.jiph.2020.06.016; https://doi.org/10.1021/acs.jproteome.0c00383; https://www.nature.com/articles/s41598-020-70863-9; https://doi.org/10.1002/minf.202000115 )

Response: Thanks for the helpful suggestion. As per your suggestion, we have added above relevant citations of methods about computational drug screening in Line 82-84 .

>>3. Fig. 1: please edit figures to make text readable (I'm no more in my 20th, but reading was hard even when wearing glasses...)

Response: Thank you. It is a very helpful suggestion. We have changed fonts size in the Figure 1, and make it more clear and readable in the revised manuscript.

>>4. Lines 118-121: the paragraph (from "Molecular" to "top hits") needs to be rephrased for better clarity.

Response: Thanks for the comment. We have carefully checked the writing in those Lines, and revised this paragraph for better clarity. For instance, previous sentence “Molecular vector-based deep learning method (DFCNN), structure feature-based deep learning method (DeepBindBC) and molecular docking (Autodock Vina) are complementary, which can be seen from Supplementary Table 2 that three methods predict different drug compounds as top hits.” is changed into “Interestingly, from Supplementary Table 2, we notice that DFCNN, DeepBindBC and Autodock Vina predict different drug compounds as top hits. This indicates that these three methods may potentially complementary to each other through judging protein-ligand interactions from different perspectives” in the Line 131-134.

>> 5. Line 123: "rejected"..."that had poor..."

6. Lines 168-169: van der Waals

7. Line 170: "metadynamics"

8. Lines 173-174: energies in kJ/mol should be rounded to one decimal place9: Lines 192-194: the paragraph (from "A recent" to "Table 1") needs to be rephrased for better clarity.

10. Line 200: "interacting....which is consist of"

11. Line 202: "it is possible that Azithromycin and Pralatrexate occupy"

12. Line 208: "activity of Azithromycin,..."

13. Line 229: I would not say that the results is "obvious" (maybe "expected")

14. Lines 275-276 "its extremely low EC50"

15. Line 334: "many electron donors and acceptors"

16. Line 367: "within I-TASSER"

17. Lines 394-404: do the reported parameters belong to the current work or are them from ref 11? Please only report relevant information

18. Line 414: "PDB"

19. Line 439: "Note that the..."

20. Lines 445-446: from "Terminals will" to "pocket" needs rephrasing

21: Line 451: "ions were added"

22. Lines 459-460: check spacing

23. Lines 467-468: "indicates that protein-lingand..."

24. Line 474: which "function"?

25. Line 574: "Thanks to TargetMol for providing"

26. Line 829. "The physico-chemical"

Response: Thanks for the helpful advices. We have corrected all above mentioned problems in the revised manuscript.

For part two of Question 8, we have changed the sentence into “In a recent study, the authors have shown that the monophosphate active form of Remdesivir interacts with the RdRp and covalently incorporated into partial double stranded RNA template of RdRp at the +1 position [7].” In Line 225-227. For Question 17, the reported results from DFCNN are using the same parameters as the current work. We have clarified this in the revised manuscript in Line 360.

**Reviewer #3**

**Comments**

1/ In the Force field based simulations 14 compounds are processed. However the free energy of only 4 compounds is given in supp table 3. Why are the other 10 compounds not listed?

2/ It would also be interesting to calculate the free binding energy of Remdesivir because it is also tested in vitro.

3/ Why are only those 4 compounds tested in vitro? what is the rationale to select only those ones?

4/ The experimental activities of the 2 compounds mentioned in the abstract do not correlate with the free energy of binding in supp table 3. Any explanation?

5/ check English + typo's: metadyanmics, ...

6/ DATA AVAILABILITY: Do not write "structure has been deposited in the PDB" but "structure has been retrieved ...". Those structures are the work of other research groups.

>>Question 1/ In the Force field based simulations 14 compounds are processed. However the free energy of only 4 compounds is given in supp table 3. Why are the other 10 compounds not listed?

Response: Thanks for the helpful question. The free energy landscapes of other 10 compounds are shown in Supplementary Fig. 4a(i). It shows that they are much favor in water than binding in the pocket. In such case, the binding free energy would be positive and we can’t accurately estimate the free energy value; it clearly indicates the ligands not prefer to binding. We have added a sentence in the Supplementary Table 3 of the revised manuscript to clarify this in the revised manuscript.

>>2/ It would also be interesting to calculate the free binding energy of Remdesivir because it is also tested in vitro.

Response: Thanks for the valuable suggestions. However, we find Remdesivir has covalent bind with the RNA primer, our method can only estimate binding free energy between non-colvalent-binding protein and ligand. Furthermore, our system not contains RNA premier, so the interaction mechanism and pattern of the Pralatrexate and Azithromycin would be different from Remdesivir. We have added more discussion about this in Line 201-205 of the revised manuscript, to make this clear.

>>3/ Why are only those 4 compounds tested in vitro? what is the rationale to select only those ones?

Response: Thanks for the helpful question. In-vitro large scale testing of compounds is labour intensive and expensive. We select those 4 compounds based on our screening pipeline. As shown in Figure 1a, we first use DFCNN, autodock Vina and DeepBindBC to preliminary screen a set of potential drugs. The using more accurate while computational expensive Molecular dynamics simulation and metadynamics simulation to further screening highly potential drug binders of RdRp. We assume the free energy calculated based on metadynamics is more reliable than other methods, although it requires relatively more computational resources. So we put this step in a last stage of screening, and finally we find only those 4 compounds have negative calculated free energy (indicate favor over binding in the pocket). That’s why we only chosen those 4 compounds for experimental validation.

>>4/ The experimental activities of the 2 compounds mentioned in the abstract do not correlate with the free energy of binding in supp table 3. Any explanation?

Response: Thanks for the helpful question. Yes, we also noticed this. The computational methods of free energy calculations include several limitations. This includes approximations involved for deriving force field parameters, and approximations in estimating energy contributions. An exact correlation between computational calculation and experimental free energy is not possible. Nonetheless, our computational calculations show good approximations when used in a screening context. We listed detailed possible reason in follow:

1. The free energy calculated by Metadynamics simulation is highly dependent on the CV we chosen, so with only one CV (coordinate number), it is possible that calculated free energy is not so accurate.
2. The MD simulation parameter currently used is still having large space to improve. So the exact binding affinity should be determined by experimental method.
3. The experimental activities may not always directly correlate with binding free energy, for instance, weak binding on critical residues may demonstrate better activity than binding on non-critical residues.

So it is quite complicated for some cases in estimating exact compound activity by purely metadynamics based free energy calculation. We have added an extra discussion about this in Line 297-305 of the revised manuscript.

>>5/ check English + typo's: metadyanmics, ...

Response: Thanks for the helpful advice. We have changed this typing error in the modified manuscript.

>>6/ DATA AVAILABILITY: Do not write "structure has been deposited in the PDB" but "structure has been retrieved ...". Those structures are the work of other research groups.

Response: Thanks for your helpful comments. We have changed the writing into “The structures for SARS-CoV-2 RdRp have been retrieved from the Protein Data Bank with accession ID 6M71, 6WTT. The template structure we used to construct RdRp-ligand complex is retrieved from the Protein Data Bank with accession ID 3BR9).”

**Problem about the data availability：**

Have all data underlying the figures and results presented in the manuscript been provided?

We have deposited all data underlying the figures and results into the github (https://github.com/haiping1010/SARS_RdRp_drug_screening_data) for the convenience of readers. The MD trajectory and some original experimental data which were too large to deposit in github were putted in figshare.com with a download link (<https://figshare.com/s/c63312c68952cdf6b586>). We added this information in the revised manuscript.
